# Eeyarestatin 24 impairs SecYEG‐dependent protein trafficking and inhibits growth of clinically relevant pathogens

**DOI:** 10.1111/mmi.14589

**Published:** 2020-09-03

**Authors:** Maurice Steenhuis, Gregory M. Koningstein, Julia Oswald, Tillman Pick, Sarah O’Keefe, Hans‐Georg Koch, Adolfo Cavalié, Roger C. Whitehead, Eileithyia Swanton, Stephen High, Joen Luirink

**Affiliations:** ^1^ Department of Molecular Microbiology Amsterdam Institute of Molecular and Life Sciences (AIMMS) Vrije Universiteit Amsterdam the Netherlands; ^2^ Institute of Biochemistry and Molecular Biology Faculty of Medicine University of Freiburg Freiburg Germany; ^3^ Experimental and Clinical Pharmacology and Toxicology Saarland University Homburg Germany; ^4^ School of Biological Sciences Faculty of Biology, Medicine and Health University of Manchester Manchester UK; ^5^ School of Chemistry Faculty of Science and Engineering University of Manchester Manchester UK

**Keywords:** eeyarestatin 1, *Escherichia coli*, nitrofurantoin, Sec61

## Abstract

Eeyarestatin 1 (ES1) is an inhibitor of endoplasmic reticulum (ER) associated protein degradation, Sec61‐dependent Ca^2+^ homeostasis and protein translocation into the ER. Recently, evidence was presented showing that a smaller analog of ES1, ES24, targets the Sec61‐translocon, and captures it in an open conformation that is translocation‐incompetent. We now show that ES24 impairs protein secretion and membrane protein insertion in *Escherichia coli* via the homologous SecYEG‐translocon. Transcriptomic analysis suggested that ES24 has a complex mode of action, probably involving multiple targets. Interestingly, ES24 shows antibacterial activity toward clinically relevant strains. Furthermore, the antibacterial activity of ES24 is equivalent to or better than that of nitrofurantoin, a known antibiotic that, although structurally similar to ES24, does not interfere with SecYEG‐dependent protein trafficking. Like nitrofurantoin, we find that ES24 requires activation by the NfsA and NfsB nitroreductases, suggesting that the formation of highly reactive nitroso intermediates is essential for target inactivation in vivo.

## Graphical abstract

ES24, but not nitrofurantoin, inhibits SecYEG‐dependent protein transport in bacteria and exhibits antibacterial activity toward various clinically relevant strains. In addition, ES24 affects many other processes important to *Escherichia coli*, hinting at a multifaceted mode of action of ES24.

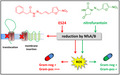

## 1 INTRODUCTION

The synthesis and trafficking of proteins that enter the secretory pathway of eukaryotic cells is coordinated at the endoplasmic reticulum (ER). Translocation of secretory proteins into the ER lumen occurs via the ER membrane‐embedded Sec61‐translocon, the core of which consists of the subunits Sec61α, Sec61β, and Sec61γ (Denks *et al*., 2014). The same complex is used for the integration of most membrane proteins. Typically, nascent pre‐secretory and membrane proteins are targeted to the translocon by the signal recognition particle (SRP) that binds to the hydrophobic signal peptide or signal anchor sequence as it emerges from the ribosome. The SRP delivers the ribosome‐nascent chain complex to the translocon in a GTP‐dependent, vectorial process that involves the membrane‐associated SRP receptor (Lang *et al*., 2017; Rapoport *et al*., 2017).

Nascent presecretory proteins enter the ER in an unfolded state and are assisted in folding by chaperones and folding catalysts. Failure to attain a native conformation due to perturbations of the folding process, mutations, or stress, may lead to a block in secretion and retention in the ER whereby misfolded secretory proteins become substrates of the ER‐associated degradation (ERAD) system (Buchberger *et al*., 2010). This is a collection of quality control mechanisms that clears the ER of accumulated, misfolded, proteins through their coordinated ubiquitination and retrotranslocation into the cytosol. The cytoplasmic AAA‐ATPase p97 utilizes the energy from ATP hydrolysis to extract the ubiquitinated proteins from the ER to be degraded by the 26S proteasome (Lemus and Goder, 2014; Ruggiano *et al*., 2014).

**FIGURE 1:**
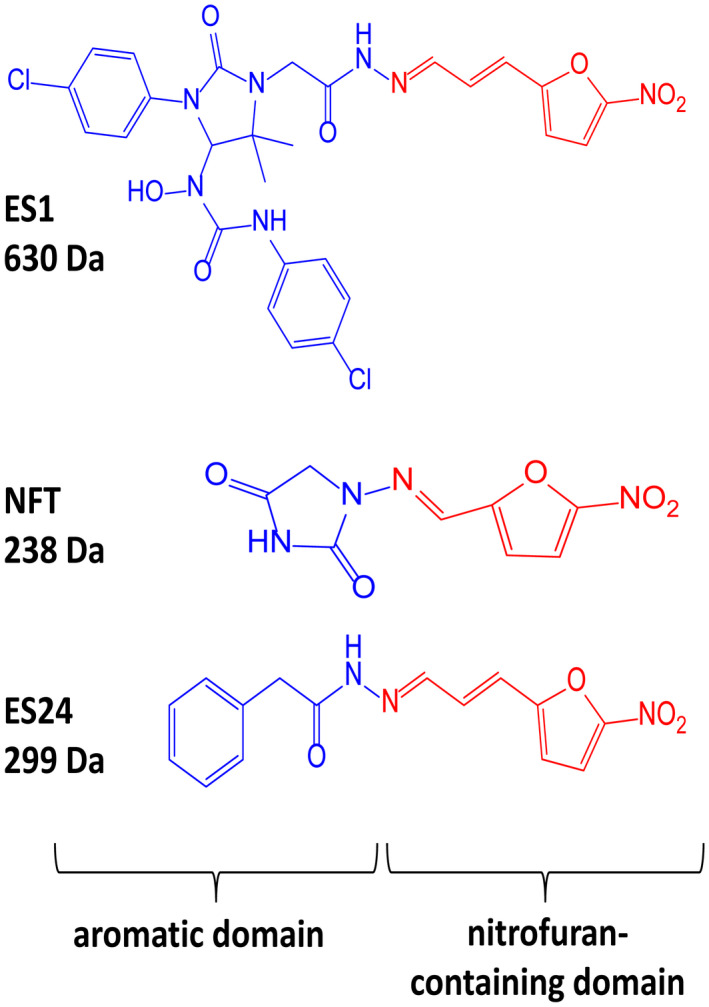
Structural formulas of ES1, ES24, and NFT. Structural formulas of ES1, ES24, and NFT with the aromatic domain shown in blue and the nitrofuran‐containing domain (NFC) in red [Colour figure can be viewed at wileyonlinelibrary.com]

Several studies have identified eeyarestatin 1 (ES1) as an inhibitor of the ERAD pathway/AAA‐ATPase p97, leading to accumulation of poly‐ubiquitinated ERAD substrates in the cell (Wang *et al*., 2008; 2010; Aletrari *et al*., 2011; McKibbin *et al*., 2012). Treatment of tumor cells with ES1 in combination with proteasome inhibitors results in proteotoxic stress and cell death, identifying ES1 as a potential anticancer drug (Sannino and Brodsky, 2017). In addition to its effect on ERAD, ES1 also inhibits the forward translocation of nascent polypeptides into the ER, most likely by a direct inhibition of the Sec61‐translocon (Cross *et al*., 2009; Aletrari *et al*., 2011; McKenna *et al*., 2016; Gamayun *et al*., 2019). ES1 is a 630 Da compound composed of a nitrofuran‐containing (NFC) domain and a separate aromatic domain (Figure 1). Recently, Gamayun et al. studied a smaller ES1 derivative of 299 Da, called ES24 (Figure 1), that closely resembles the NFC‐domain of ES1. They found that ES24 retained the ability to inhibit Sec61‐dependent protein translocation into ER derived rough microsomes to a similar extent as ES1 and proposed that both ES1 and ES24 bind to the lateral gate of the Sec61‐translocon in such a way as to lock it into an open state that is still Ca^2+^ permeable but incompetent for protein translocation (Gamayun *et al*., 2019). In contrast to ES1, ES24 does not interfere with cellular deubiquitination pathways, most likely because the presence of the aromatic domain of ES1 is required to inhibit AAA‐ATPase p97 (Wang *et al*., 2010).

The trimeric SecYEG‐translocon in the bacterial cytoplasmic membrane is homologous to the eukaryotic Sec61‐translocon and mediates the translocation of proteins across, and their insertion into, this membrane (Denks *et al*., 2014). Given its structural and functional conservation we considered the possibility that ES1 and ES24 may also inhibit SecYEG‐dependent protein transport in bacteria and, given the essential nature of this process, thereby exhibit antibacterial activity. Focusing on *Escherichia coli* as a model organism, we showed that ES1 was unable to cross the outer membrane due to its size. In contrast, its smaller derivative ES24 impaired SecYEG‐mediated protein transport and membrane insertion, inducing a stress response and affecting growth. Transcriptomic analysis revealed that ES24 either directly or indirectly affects several other cellular processes important to *E. coli*, suggesting that ES24 has additional targets and a multifaceted mode of action. Finally, ES24 showed antibacterial activity toward various clinically relevant bacterial strains that was equivalent to or stronger than the activity of nitrofurantoin, a known antibiotic and structural homolog of ES24.

## 2 RESULTS

### 2.1 ES24 inhibits growth of *E. coli*

ES1 and its smaller derivative ES24 inhibit Sec61‐mediated protein translocation across the ER membrane (Cross *et al*., 2009; Gamayun *et al*., 2019). To examine the effect of these compounds on the equivalent prokaryotic SecYEG‐translocon, we started by evaluating the effect of ES1 and ES24 on cell viability with *E. coli* as a model organism. Since the SecYEG‐translocon is essential, compromising its function is expected to affect growth (Du Plessis *et al*., 2011). To test this, *E. coli* strains were grown in liquid culture in the presence or absence of ES1 and ES24 to determine the minimal inhibitory concentration (MIC). As shown in Table 1, ES24 strongly affected growth of the wild‐type MC4100 strain with a MIC of 25 µM, whereas ES1 showed no effect up to the highest concentration tested (100 µM). Importantly, ES1 has a molecular mass of 630 Da, which may hamper its passage through the outer membrane that has a molecular sieve function with an exclusion limit of around 600 Da (Nikaido, 2003), potentially explaining why ES1 does not affect growth. In contrast, the outer membrane does not apparently obstruct passage of the smaller ES24 (299 Da) from accessing the cell.

**TABLE 1:** Overview of the MIC (µM) of ES1, ES24, and NFT against the indicated bacterial strains

Strain	ES1 (630 Da)^a^	ES24 (299 Da)^a^	NFT (238 Da)^a^
*E. coli* MC4100	>100	25	25
*E. coli* MC4100 pABCON2‐*fhuA ∆C/∆4L*	25	12	25
*E. coli* AB1157	>100	25	25
*E. coli* NER502 ∆*nfsA* and ∆*nfsB*	>100	>100	>100

^a^ Inhibitory curves can be found in the supporting information (Figure S1).

To investigate the potential influence of outer membrane permeability, we used an *E. coli* strain that expresses a genetically modified variant of the large outer membrane β‐barrel protein FhuA that lacks its N‐terminal plug domain and four large external loops (Krishnamoorthy *et al*., 2016). This so called FhuA ∆C/∆4L has a predicted pore size of 2.4 nm and allows unhindered passage of molecules up to ~2,000 Da (Niedzwiecki *et al*., 2012; Krishnamoorthy *et al*., 2016). As shown in Table 1, this so‐called hyperporination of *E. coli* cells allowed ES1 to affect growth with a MIC of 25 µM, similar to that for ES24 in the parental *E. coli* strain. We therefore conclude that under normal circumstances the outer membrane does indeed restrict the entry of ES1. Interestingly, the FhuA ∆C/∆4L strain also displayed a modest increase in sensitivity to ES24 indicative of the more efficient uptake of this compound as well as the larger ES1.

### 2.2 ES24 requires activation by nitroreductases to affect cell growth

The antibiotic nitrofurantoin (NFT), which is used as a front‐line treatment of urinary tract infections, contains the same NFC‐domain as ES24, and is thus structurally related (Figure 1) (Huttner *et al*., 2015). The NFC‐domain can be reduced by nitroreductases via a highly reactive nitroso intermediate, causing damage to proteins and DNA via nucleophilic reactions and redox chemistry (Sandegren *et al*., 2008). Indeed, it has been shown that reduction of the nitro group of NFT by the nitroreductases NfsA and NfsB is required for its effect on bacterial growth through damaging DNA, RNA and proteins (Whiteway *et al*., 1998; Valle *et al*., 2012). To investigate whether ES24 also requires activation by nitroreductases, its effect on growth was evaluated in a double ∆*nfsA* and ∆*nfsB* mutant strain. Similar to NFT, ES24 did not affect growth in the mutant strain up to the highest concentration tested (100 µM), whereas growth in the parental wild‐type strain was affected with a MIC of 25 µM for both compounds (Table 1). Apparently, and analogous to NFT, reduction of the NFC‐domain is required for the effect of ES24 on cell growth.

### 2.3 ES24 inhibits SecYEG‐mediated insertion of cytoplasmic membrane proteins

**FIGURE 2:**
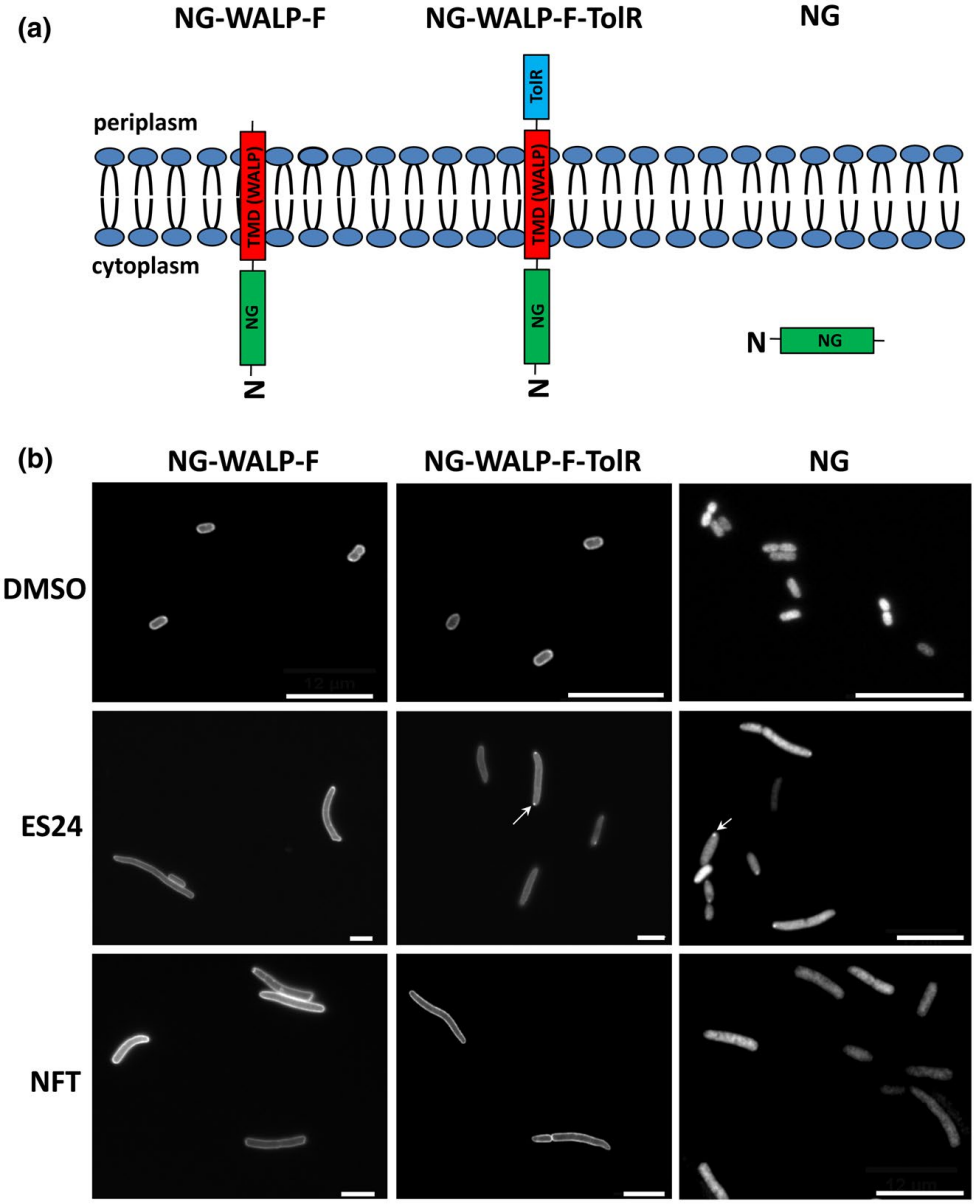
ES24 impairs membrane insertion of NG‐WALP‐F‐TolR. (a) Schematic representation of the topology of the NG‐WALP‐F, NG‐WALP‐F‐TolR and NG constructs. (b) *E. coli* MC4100 cells, harboring pSE(p15a)‐NG, pSE(p15a)‐NG‐WALP‐F or pSE(p15a)‐NG‐WALP‐F‐TolR, were grown in a 96‐well plate and incubated for 1 hr with either 0.25× MIC ES24, NFT or DMSO as control. Protein expression was then induced with IPTG for 1 hr before cells were fixed and analyzed by fluorescence microscopy. Images were analyzed by ImageJ using the Coli‐Inspector plugin. A representative image per condition is shown. Potentially aggregated NG is indicated by an arrow. Scale bars are 10 µm. Quantification and statistical analysis can be found in the supporting information (Figures S2 and S3) [Colour figure can be viewed at wileyonlinelibrary.com]

We considered the possibility that the effect of ES24 on cell viability in *E. coli* may correlate with its targeting of the essential SecYEG‐translocon. To address this possibility directly, we examined the effect of ES24 on the membrane localization of previously defined SecYEG‐dependent and SecYEG‐independent inner membrane proteins (IMPs). For this, we used two synthetic bitopic IMPs consisting of a cytoplasmic NeonGreen (NG) reporter domain, a WALP transmembrane domain (TMD) of moderate hydrophobicity and a C‐terminal periplasmic domain of two different lengths (Figure 2a). WALPs are peptides that consist solely of tryptophan (W), alanine (A) and leucine (L) residues and can serve as synthetic TMDs of distinct hydrophobicity (Holt and Killian, 2010; Peschke *et al*., 2019). We have shown previously that the construct NG‐WALP‐F with a periplasmic domain of only two amino acids behaves as a tail‐anchored protein that enters the inner membrane posttranslationally in a SecYEG‐independent mechanism (Peschke *et al*., 2019). In contrast, NG‐WALP‐F‐TolR, which carries the 102‐residue periplasmic domain of the type‐II IMP TolR, requires SecYEG for membrane insertion in a process that is believed to occur concomitant with translation (Peschke *et al*., 2019).

The presence of NG in the synthetic constructs allows reliable monitoring of membrane association by the formation of a halo‐type staining of cell envelopes in fluorescence microscopy. *E. coli* cells harboring NG‐WALP‐F, NG‐WALP‐F‐TolR, or control NG‐expression constructs where NG is located in the cytoplasm, were grown for 1 hr in the presence of 0.25× MIC of ES24, NFT or DMSO as a control, before reporter protein expression was induced with isopropyl β‐d‐1‐thiogalactopyranoside (IPTG). After 1 hr of continued growth, cells were collected, fixed and analyzed by fluorescence microscopy. DMSO‐ or NFT‐treated cells expressing the reporter constructs showed disperse circumferential labeling indicative of membrane localization (Figure 2b) (Peschke *et al*., 2019). In contrast, ES24 prevented membrane localization of the SecYEG‐dependent NG‐WALP‐F‐TolR reporter but not of the SecYEG‐independent NG‐WALP‐F. NG expression and its localization in the cytosol were not altered by ES24 and NFT, as expected (Figures 2b and S3). Of note, both ES24 and NFT caused significant cell elongation independent of which WALP protein was expressed, most‐likely as a consequence of their overall toxic effect on the cells. We also noted that cells expressing NG and NG‐WALP‐F‐TolR exhibited fluorescent spots at the cell poles indicating either the aggregation of mislocalized WALP proteins or their recruitment to pre‐existing aggregates, consistent with impaired function of the SecYEG‐translocon (Peschke *et al*., 2019). We additionally analyzed the fluorescence distribution in individual cells by creating cross‐sectional profiles of the cells and assessing membrane localization based on the center (cytosol) to border (membranes) ratio of the fluorescent signal (Peschke *et al*., 2019). This semi‐quantitative analysis (Figure S2a,b) confirmed the conclusions that we made based on our qualitative visual inspection.

Together, these data strongly suggest that ES24 directly or indirectly inhibits the insertion of SecYEG‐dependent membrane proteins. In contrast, the antibiotic NFT that is structurally related to ES24 does not appear to interfere with SecYEG‐mediated membrane insertion.

### 2.4 ES24 inhibits secretion of Hbp

**FIGURE 3:**
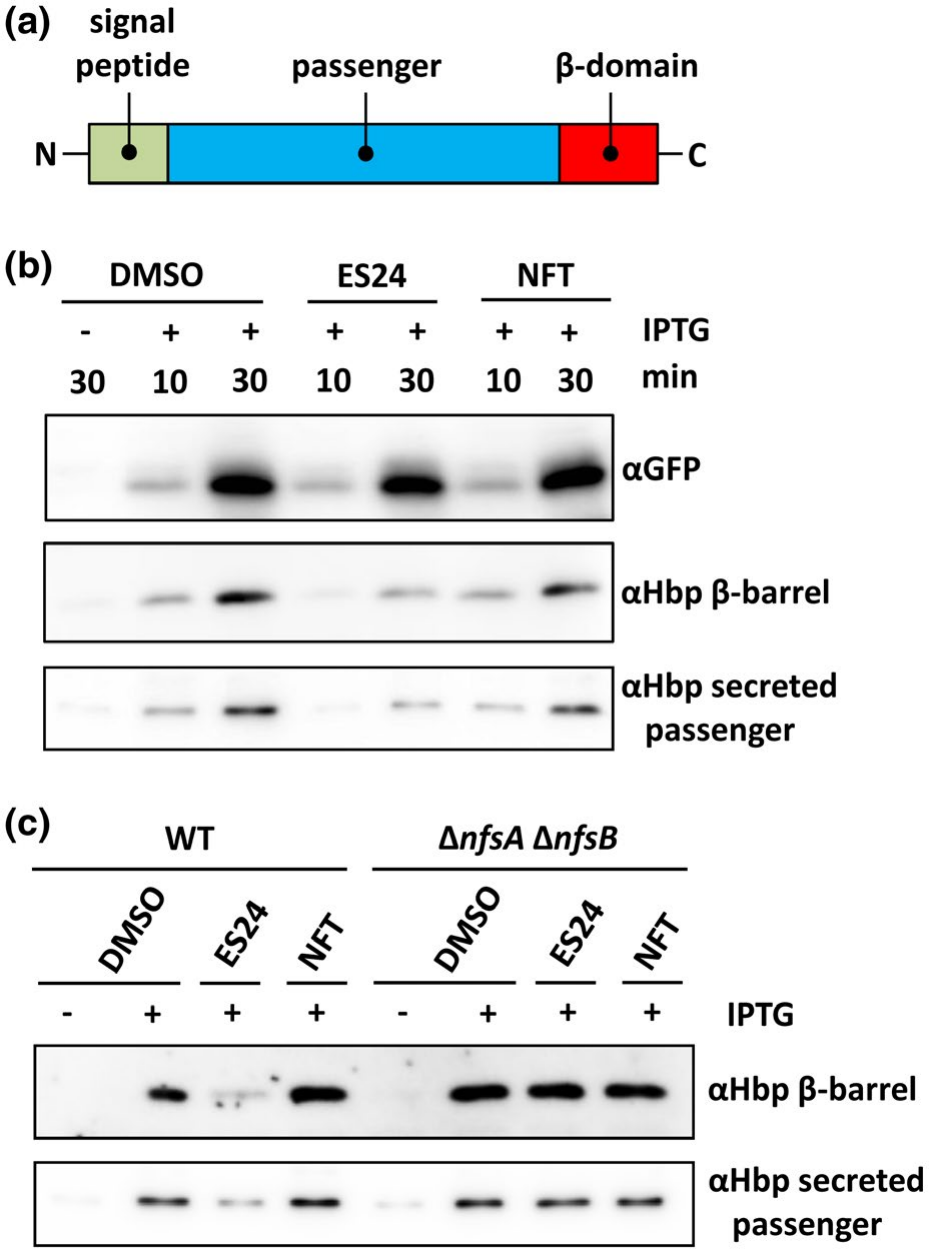
ES24 impairs Hbp secretion and requires reduction by NfsA/B to impose this effect. (a) Schematic overview of the domain organization of the autotranporter Hbp. (b) *E. coli* TOP10F′ bacteria were grown in a round bottom tube and exposed to 0.5× MIC ES24 and NFT or DMSO as control. Hbp and GFP were expressed from pEH3 using IPTG for induction. After 10 and 30 min of exposure whole cell lysates were analyzed by SDS‐PAGE and Western blotting using antibodies against GFP and Hbp β‐barrel. The spent medium was TCA precipitated and analyzed by Western blotting using antibodies against the Hbp passenger domain. (c) *E. coli* NER502 ∆*nfsA/B* and its wild‐type parental strain were grown as described above and exposed to 0.5× MIC ES24 and NFT or DMSO as control. After 30 min of Hbp expression from pEH3, using IPTG for induction, whole cell lysates were analyzed by SDS‐PAGE and Western blotting using antibodies against the Hbp β‐barrel domain. The spent medium was TCA precipitated and analyzed by Western blotting using antibodies against the Hbp passenger domain. Data are representative of three independent experiments. Samples were corrected for growth (OD_600_) in order to load equal amounts [Colour figure can be viewed at wileyonlinelibrary.com]

To obtain independent evidence that the function of the SecYEG‐translocon is affected by ES24, we analyzed secretion of the autotransporter hemoglobin protease (Hbp). Hbp is synthesized in the cytosol and consists of three domains: a signal sequence, a passenger domain and a C‐terminal β‐domain (Figure 3a) (Jong *et al*., 2010; van Ulsen *et al*., 2013). Hbp is transported to the periplasm by the SecYEG‐translocon, where the signal peptide is cleaved and the so‐called pro‐Hbp interacts with periplasmic chaperones that support targeting to the BAM complex in the outer membrane (Sijbrandi *et al*., 2003; Sauri *et al*., 2009). The β‐domain then folds into a β‐barrel structure assisted by the BAM complex and the passenger is secreted across the outer membrane and cleaved from its β‐barrel to be released into the extracellular environment. To examine whether ES24 affects Hbp secretion, *E. coli* cells containing an Hbp expression construct were grown and induced for Hbp expression in the presence or absence of ES24, NFT, or a DMSO control. To monitor secretion over time, samples were withdrawn after 10 and 30 min followed by SDS‐PAGE and Western blotting (Figure 3b). In the control DMSO‐treated sample, Hbp was processed and secreted, as shown by the presence of the 110 kDa secreted Hbp passenger in the supernatant and the 30 kDa cleaved β‐domain in the cell pellet, which was already detectable after 10 min and increased in intensity at the 30 min time point. Treatment with ES24 led to a decrease in the amount of secreted passenger in the supernatant with a corresponding decrease in the amount of the cleaved β‐domain in the cell pellet, while NFT did not affect Hbp processing and secretion when compared to DMSO‐treated cells. To exclude a possible effect on gene expression, *gfp* was cloned in the same pEH3 vector and, as shown in Figure 3b, neither ES24 nor NFT affected its expression in this context.

Since we had established that reduction of ES24 by NfsA and NfsB is required to inhibit cell growth we were interested as to whether reduction is also required for the ES24‐mediated inhibition of SecYEG function. To test this, we monitored expression and secretion of Hbp in the double ∆*nfsA* and ∆*nfsB* mutant strain. Incubation with ES24 resulted in impaired Hbp secretion in the parental strain, but not in the deletion mutant (see Figure 3c), indicating that a reduced form of ES24 is required for the inhibition of SecYEG‐dependent protein secretion.

Taken together, we conclude that ES24 specifically inhibits protein insertion into and translocation across the inner membrane via the SecYEG‐translocon, arguing that this protein conducting channel is a potential target of this compound.

### 2.5 NFT does not elicit Sec61 dependent Ca^2+^ leakage from the ER

**FIGURE 4:**
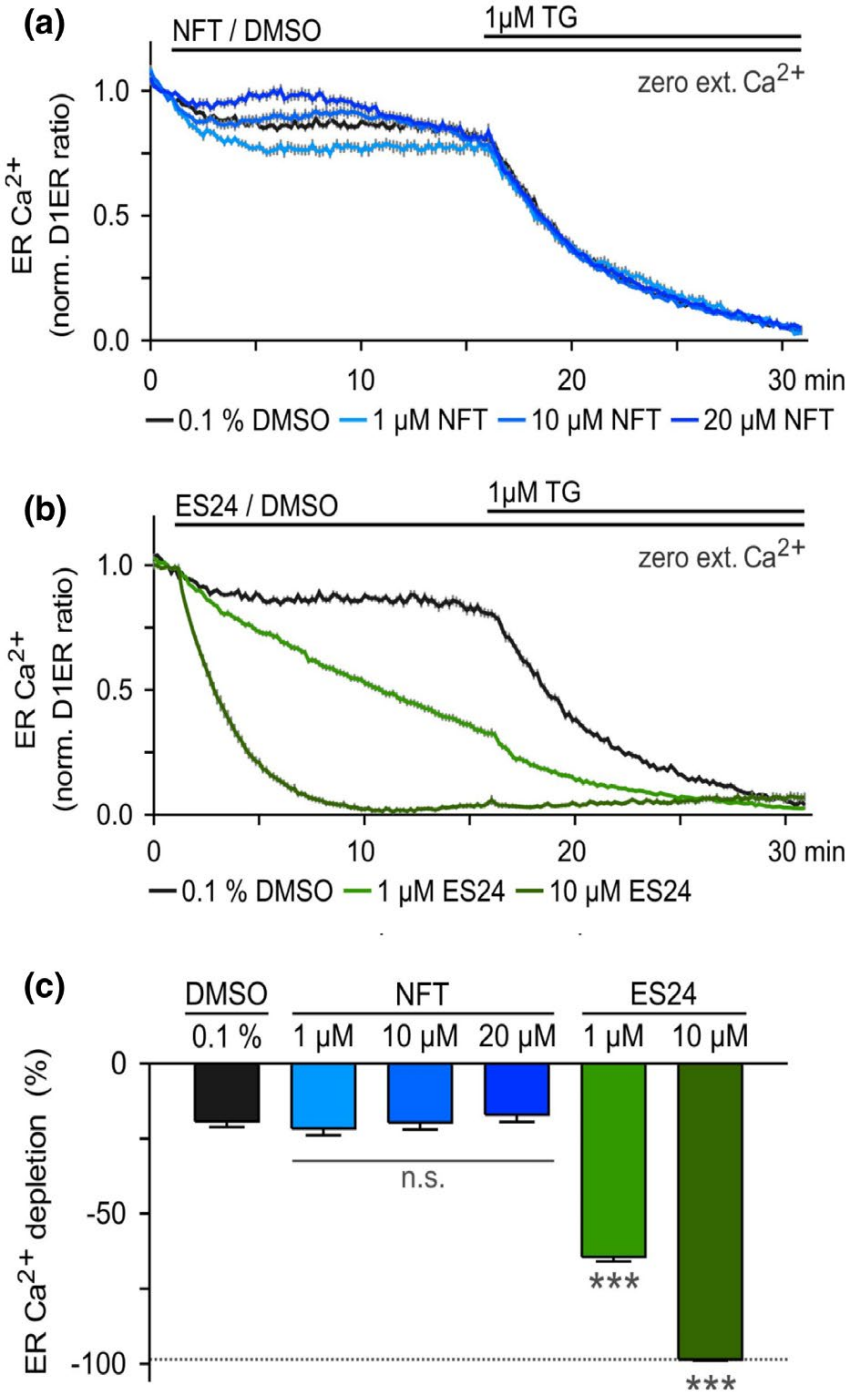
NFT does not cause Ca^2+^ leakage from ER. Changes in the Ca^2+^ concentration of the ER were monitored with the Ca^2+^ sensor D1ER expressed in HEK cells. Cells were exposed to various concentrations of (a) NFT and (b) ES24 for 15 min to explore possible changes in ER Ca^2+^ when SERCA pumps were active. Graphs depict ER Ca^2+^ expressed as normalized D1ER ratios. Subsequently, SERCA pumps were blocked with 1 µM thapsigargin (TG) to unmask the Ca^2+^ leakage from ER. (c) Comparison of the ER Ca^2+^ depletion between DMSO controls and NFT‐ or ES24‐treated cells. ER Ca^2+^ depletion was measured 1 min before TG‐application. The dotted line illustrates the maximal Ca^2+^ depletion at 30 min. N: 17–34 cells per experimental setting. n.s., non‐significant; ***, *p* < .001 [Colour figure can be viewed at wileyonlinelibrary.com]

Gamayun and coworkers have shown that ES24 provokes the leakage of Ca^2+^ from the ER, an effect that was proposed to be caused by its interaction with the lateral gate of Sec61α that captured it in an open, Ca^2+^ permeable, but protein translocation‐incompetent conformation (Gamayun *et al*., 2019). To further explore the specificity of ES24 for conserved membrane translocation complexes, we used Sec61 mediated Ca^2+^ leakage as a proxy for channel function and compared the effect of ES24 and NFT using the fluorescence‐resonance‐energy‐transfer‐based (FRET) Ca^2+^ sensor D1ER expressed in HEK cells (Palmer *et al*., 2004; Gamayun *et al*., 2019). A change in FRET signal thus reflects changes in the amount of Ca^2+^ in the ER (Palmer *et al*., 2004). As shown in Figure 4a, exposure of HEK‐D1ER cells to up to 20 µM NFT failed to produce an effect that could be distinguished from the natural variations in level of ER Ca^2+^ in DMSO‐treated control cells observed during the initial 15 min of these experiments. In contrast, and consistent with earlier data, ES24 caused a rapid reduction in ER Ca^2+^ content, even at a concentration of 1 µM, during the first 15 min of exposure; while the effects at 10 µM ES24 were even more dramatic, producing full ER Ca^2+^ depletion within 7–9 min exposure as judged by the total loss of FRET signal (Figure 4b) (Gamayun *et al*., 2019). Looking at ER Ca^2+^ depletion after 14 min of compound exposure, we can clearly see a dramatic effect of ES24, but no significant effect of NFT when compared to control DMSO‐treated cells (Figure 4c). Subsequently, SERCA pumps were blocked with 1 µM thapsigargin (TG) to prevent Ca^2+^ influx and thus unmask the Ca^2+^ leakage from ER. The time courses of the TG‐induced ER Ca^2+^ depletion were similar in cells exposed to DMSO and NFT (decay time constants: 340.22 s, DMSO; 323.56–382.36 s. NFT), indicating that NFT did not modify Ca^2+^ leakage from ER. The time course of the TG‐induced ER Ca^2+^ depletion with 1 µM ES24 was slightly faster than that of cells treated with DMSO (340.22 s, DMSO; 301.28 s, 1 µM ES24), confirming increased Ca^2+^ leakage during ES24 treatment (Figure 4a,b). Thus, these Ca^2+^ flux experiments strongly suggest that the impaired ER Ca^2+^ homeostasis is due to specific structural features of ES24 that distinguish it from its close relative NFT.

### 2.6 ES24 and NFT induce a broad range of stress responses

**FIGURE 5:**
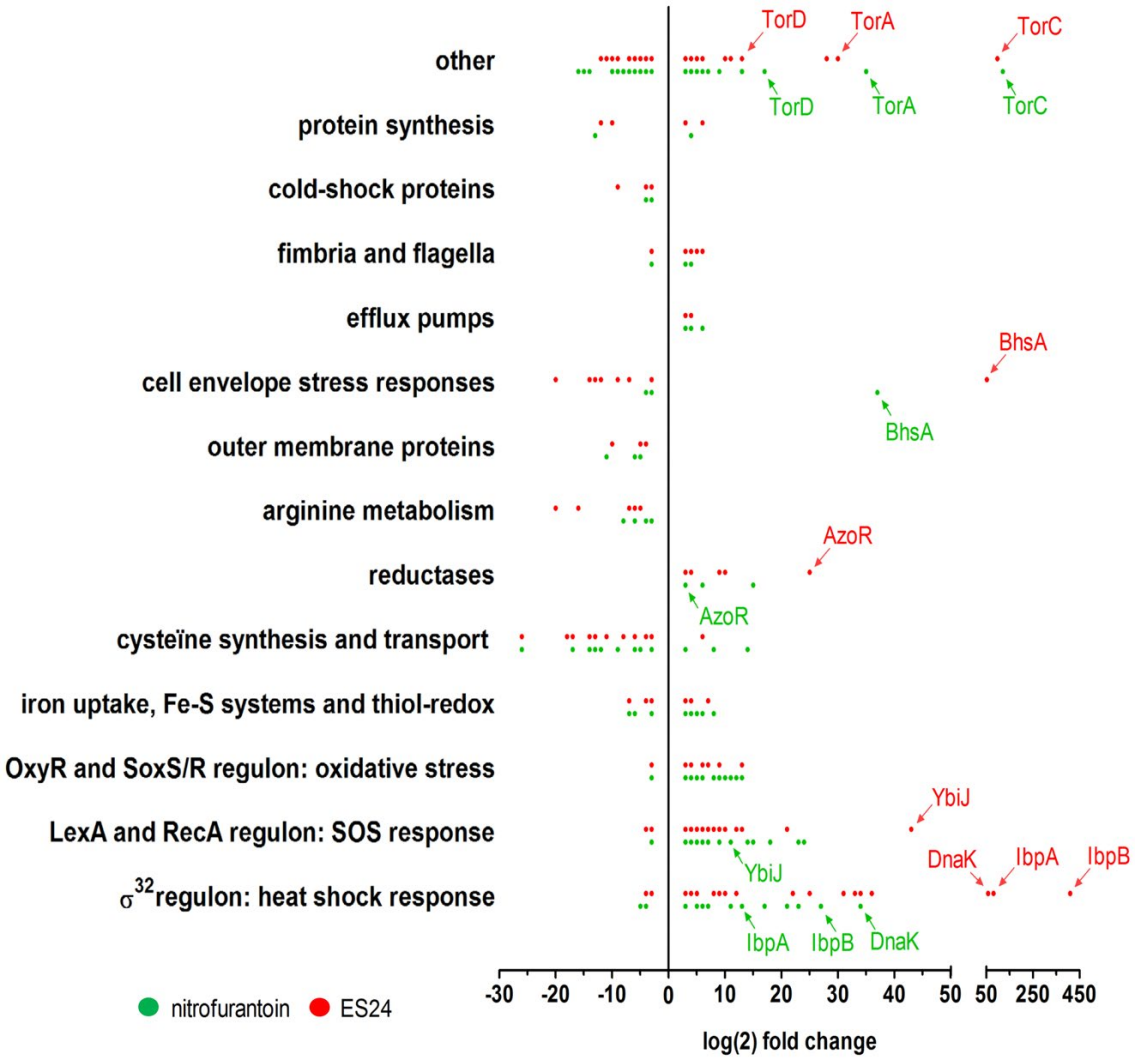
NFT and ES24 induce a broad range of stress responses. Transcriptomic profile of differentially expressed genes in *E. coli* MC4100 treated with either 0.5× MIC ES24 (red dots) or NFT (green dots) compared to DMSO‐treated cells. Indicated are the transcripts that showed a log(2) fold‐change of ≥3 (*p* < .05). Genes are clustered into functional groups or regulons with each dot representing a gene, of which some are annotated. See supporting information for full transcriptomic analysis (Table S3) [Colour figure can be viewed at wileyonlinelibrary.com]

The presence of the NFC‐domain in ES24 that is reduced via highly reactive nitroso intermediates, may imply that targets other than SecYEG are also affected by ES24, as has been suggested for NFT (McOsker *et al*., 1994). To obtain unbiased insight into the cellular responses elicited by both compounds, we performed global gene‐expression profiling using RNA sequencing. *E. coli* MC4100 cells were treated with ES24 or NFT at 0.5× MIC or DMSO as control for 15 min and total RNA was isolated and analyzed. A total of 195 and 191 genes were either upregulated or downregulated by at least 3‐fold (*p* ≤ .05) upon treatment with ES24 and NFT, respectively, with 55 of these genes found to be differentially expressed between the two compounds (Figure 5). For both compounds, we observed that a number of stress‐related genes were induced, in particular those belonging to the heat‐shock, SOS‐ and oxidative stress regulons, which is in line with the expected damage caused by nitroso intermediates. Many genes of the σ^32^ (heat shock)‐regulon were highly upregulated, including several genes coding for molecular chaperones or proteases. The master regulators of the SOS‐response, *lexA*, and *recA*, were upregulated as well as the corresponding regulon members involved in DNA repair. Many genes that belong to the SoxR/SoxS and OxyR oxidative defense regulons were also highly upregulated; including those encoding catalase‐peroxidase (*katG*), superoxide dismutase (*sodA*), enzymes involved in redox homeostasis (*grxA*, *gshA*, and *gshB*) and many factors involved in formation and repair of Fe‐S clusters (*iscARSU* and *sufABCDES* operons). Cysteine and arginine synthesis were downregulated following treatment with both ES24 and NFT. Interestingly, the reductases *nfsA* and *nfsB* that are required to activate ES24 and NFT were both upregulated, as were the genes coding for the reductases AzoR and NemA. Furthermore, drug efflux pumps and the biosynthesis of flagella components appeared to be downregulated by both ES24 and NFT. The major cell envelope stress responses σ^E^, Cpx, and Psp were downregulated for both compounds. Strikingly, induction of the σ^32^‐regulon was much stronger in ES24‐treated cells as compared to NFT. In particular, *ibpA* and *ibpB* showed a 4‐fold and 15‐fold upregulation in ES24‐ vs NFT‐treated cells, respectively. IbpA (inclusion body protein A) together with IbpB associate with aggregated proteins to stabilize and protect them from proteolysis and promote refolding (Kuczyńska‐Wiśnik *et al*., 2002). Possibly, the upregulation of these components that we observe reflects the accumulation of pre‐secretory and cytoplasmic membrane proteins that accumulate in the cytosol due to inhibition of SecYEG‐translocon function by ES24.

To confirm that ES24 results in stronger activation of the heat‐shock response than NFT, we used an independent reporter assay which makes use of the gene for the fluorescent protein NG placed under control of the heat‐shock regulated *groES* promoter (Steenhuis *et al*., 2019). *E. coli* cells harboring this reporter construct were grown and incubated with a twofold dilution range of ES24 or NFT. As shown in Figure S4, ES24 induced a much stronger increase in fluorescence than NFT, confirming the specific activation of a heat‐shock stress response by ES24. As a control, we analyzed activation of the cell envelope σ^E^ response by using a similar P*rpoE*‐mNG reporter construct (Steenhuis *et al*., 2019). In agreement with the RNAseq analysis, neither NFT nor ES24 induced fluorescence in cells harboring pUA66‐RpoE‐mNG, confirming that both compounds do not provoke cell envelope stress.

In summary, ES24 and NFT induce similar responses in *E. coli* cells including heat‐shock, SOS and oxidative stress. However, ES24 has a stronger effect on the expression of specific heat‐shock proteins that may reflect cytosolic protein accumulation due to impaired SecYEG‐translocon activity.

### 2.7 Structure‐activity analysis for ES24

To examine the relevance of specific structural features of ES24 in more detail, we tested compounds with structures closely resembling ES24, together with NFT, for their effect on *E. coli* growth and the localization of the SecYEG‐dependent membrane protein NG‐WALP‐F‐TolR. The structure of ES24 consists of the 5‐nitrofuranyl‐group (Table 2; indicated in black and green), linked on the 2‐position via two conjugated trans‐double bonds (red) to a phenyl‐substituted (teal) acylhydrazide (pink). It differs from NFT in its hydrazide substituent and in the length of the central double bond spacer, while the 5‐nitrofuranyl group is retained.

**TABLE 2:** ES24 structure‐activity analysis

Compound	Structural formula^a^	MIC *E. coli* ^b^	LD_50_ HEK293^b^	Interference NG‐WALP‐F‐TolR insertion^c^
NFT	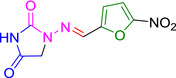	25	80	No
Furagin	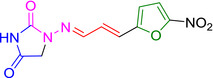	3	>80	No
ES24	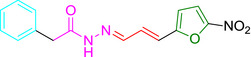	25	80	Yes
ES24‐D1	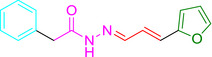	>100	>80	No
ES24‐D2	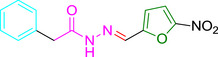	50	80	No

^a^ Structural differences between compounds are highlighted with different colors.

^b^ The MIC value and median lethal dose (LD_50_) are given in µM. Inhibitory curves can be found in the supporting information (Figure S5).

^c^ Compounds were assayed for their ability to interfere with membrane insertion of NG‐WALP‐F and NG‐WALP‐F‐TolR, as described in the legend of Figure 2. Representative microscopy pictures are shown in the supporting information (Figure S6).

As detailed above (cf. Table 1), the nitro group of NFC‐domains can be reduced by NfsA/B via a highly reactive nitroso intermediate and we find that the reduction of ES24 is required to impair bacterial growth and Hbp secretion (Figure 3). To further assess the importance of the NFC‐domain, we tested derivative ES24‐D1 that lacks the nitro substituent. This compound did neither inhibit the growth of *E. coli* nor did it affect membrane insertion of NG‐WALP‐F‐TolR (Table 2 and Figures S5 and S6), confirming the importance of the nitro group to the activity of ES24.

The central pair of conjugated trans double bonds in ES24 significantly changes the spacing between the nitrofuran and hydrazide group with respect to NFT. To investigate this feature in more detail, we tested derivative ES24‐D2, which comprises a single trans double‐bonded spacer (Table 2; in red). ES24‐D2 was still able to affect growth with a MIC of 50 µM, but did not alter membrane localization of NG‐WALP‐F‐TolR, indicating that the presence of two conjugated trans double bonds is required for the inhibitory effect on the SecYEG‐translocon. This is likely due to the increased spacer length of ES24 compared to ES24‐D2, which may affect its selectivity and reactivity with regard to SecYEG binding. The pronounced effect of the two conjugated double bonds prompted us to investigate whether a derivative of NFT, furagin that also comprises a pair of trans double bonds, is able to target the SecYEG‐translocon. Although furagin was not able to interfere with membrane insertion of NG‐WALP‐F‐TolR, its MIC is ~8‐fold lower than that for NFT suggesting that this specific linker improves the antibacterial effect. This also suggests that the observed lack of SecYEG selectivity of furagin may be related to its acylhydrazide substituent when compared to the structure of ES24 (Table 2; pink).

Finally, the compounds were tested for toxicity against mammalian HEK293 cells and all found to have a lethal median dose (LD_50_) of 80 μM or higher (Table 2). In the case of ES24, this value is consistent with a recent study that showed only mild effects on INS‐1 and NALM‐6 cells, and no effects on HEK and HeLa cells, at concentrations of up to 20 μM (Gamayun *et al*., 2019).

### 2.8 ES24 inhibits the growth of various pathogens

ES24 strongly inhibits growth of *E. coli* K12 strains and is no more toxic to HEK293 cells than the established antibiotic NFT, raising our interest in the susceptibility of pathogens to ES24. To address this, the MIC of ES24 against six pathogens that belong to the ESKAPE group (*Enterococcus faecium*, *Staphylococcus aureus*, *Klebsiella pneumoniae*, *Acinetobacter baumannii*, *Pseudomonas aeruginosa* and *Enterobacter cloacae*) (Boucher *et al*., 2013) responsible for the majority of nosocomial infections worldwide, was determined. As shown in Table 3, ES24 and NFT showed little if any toxicity against the Gram‐negative strains *K. pneumoniae*, *A. baumannii*, *P. aeruginosa*, and *E. cloacae* up to the highest concentration tested (100 µM). However, ES24 showed a much stronger growth inhibitory activity toward the Gram‐positive methicillin‐resistant *S. aureus* (MRSA) and vancomycin‐resistant *E. faecium* with a MIC of 6 and 50 µM, respectively. In both cases this activity is fourfold more potent than that of NFT.

**TABLE 3:** MIC (µM) of ES24 and NFT against the indicated bacterial strains

Strain	ES24^a^	NFT^a^
ESKAPE group		
*Enterococcus faecium* VRE	50	200
*Staphylococcus aureus* MRSA	6	25
*Klebsiella pneumoniae*	>100	>100
*Acinetobacter baumannii*	100	>100
*Pseudomonas aeruginosa*	>100	>100
*Enterobacter cloacae*	100	>100
Others		
*Enterococcus faecalis* VRE	12	100
*Bacillus subtilis* 168	1	25
*Escherichia coli* (uropathogenic)	30	30

^a^ Inhibitory curves can be found in the supporting information (Figure S7).

To further investigate the effect of ES24 on growth of Gram‐positive bacteria, we determined its MIC against vancomycin‐resistant *Enterococcus faecalis* and soil bacterium *Bacillus subtilis*. Once again, ES24 was substantially more effective than NFT against these two strains, showing an 8‐fold and 25‐fold decrease in MIC values, respectively. Perhaps these findings reflect the lack of an outer membrane which allows ES24 to penetrate Gram‐positive cells more easily. Finally, since NFT is currently used to treat urinary tract infections caused by *E. coli*, we also determined the effects on growth of an uropathogenic *E. coli* strain. As shown in Table 3, both NFT and ES24 inhibited growth with a MIC of 30 µM. Thus, ES24 exhibits potent antibacterial activity toward several pathogens, including vancomycin‐resistant *Enterococcus* spp. (VRE), MRSA and uropathogenic *E. coli*.

## 3 DISCUSSION

We have identified and characterized the inhibitory effect of ES24, member of the eeyarestatin group of small molecule inhibitors, on the activity of the membrane‐embedded *E. coli* SecYEG‐translocon using a variety of approaches. SecYEG is an attractive drug target that fulfills an essential role in the translocation and membrane integration of many proteins (Rapoport *et al*., 2017). Consistent with its recently established inhibitory effect of ES24 on protein translocation via the eukaryotic Sec61‐translocon we find that the conserved prokaryotic SecYEG‐translocon is also a likely target of ES24. Furthermore, we find that ES24 inhibits the growth of both *E. coli* and a variety of clinically relevant strains, displaying MIC values that are the same as or lower than NFT, a known antibiotic and structural analog of ES24.

ES24, but not ES1, strongly impaired growth of a wild‐type *E. coli* K12 strain upon addition to the culture medium. This difference is most likely caused by the larger size of ES1 and its consequent inability to pass the outer membrane and reach its intracellular target(s). Hence, sensitivity to ES1 was observed in a strain with a more permeable outer membrane due to the expression of a “plug‐less” FhuA pore. This strain is known to be sensitive to large‐scaffold antibiotics such as vancomycin, which is exclusively used to fight Gram‐positive pathogens (Krishnamoorthy *et al*., 2016). In line with this observation, ES1 was as active as ES24 in reducing the growth of the Gram‐positive bacterium *B. subtilis* (Figure S8).

ES24 clearly affected, either directly or indirectly, both SecYEG‐dependent protein secretion and membrane insertion. Furthermore, we show that deletion of the *nfsA/B* reductases negates the effects of ES24 on protein secretion and cell growth, which likely reflects the inability of these mutants to reduce the NFC‐domain of ES24 via a highly reactive nitroso intermediate. In further support of this hypothesis, we show that the derivative ES24‐D1 that lacks the nitro group, was unable to inhibit either growth or SecYEG‐dependent membrane protein insertion. Intriguingly, ES24 structurally resembles the known antibiotic NFT that is in clinical use to treat urinary tract infections (Huttner *et al*., 2015). NFT has a similar stretched backbone with a NFC‐domain (Figure 1) and also appears to be activated by reduction as most resistant strains were shown to carry mutations in *nfsA/B* (McCalla *et al*., 1978; Sandegren *et al*., 2008). Strikingly, although NFT has been in clinical use for decades, its mode of action and targets are not fully defined. Currently available evidence indicates that the reactive reduced intermediates that are generated by the cell interfere with ribosomes and other macromolecular targets and thereby inhibit multiple critical processes such as translation, DNA replication, and various metabolic pathways (Huttner *et al*., 2015). This multitude of targets might also explain why NFT is relatively insensitive to the development of resistance other than via the reductase mutations mentioned above (Sandegren *et al*., 2008).

An unbiased exploration by global transcriptomics analysis revealed that early after the addition of ES24 and NFT the cells reacted with a largely similar response profile. Induction of oxidative stress under control of the OxyR and SoxS/R regulons and an upregulated SOS response are consistent with the generation of highly reactive species as a result of conversion of both compounds. For NFT these responses were anticipated, though previously not shown in an unbiased analysis, and imply a generic disruptive effect of the toxic intermediates that is shared between NFT and ES24 (Tu and McCalla, 1975). Both compounds also induce genes involved in the response to heat shock stress, but here the magnitude of the response was much higher in cells incubated with ES24, as illustrated by the highly upregulated expression of inclusion body proteins A and B (IbpA and IbpB). In this case, we speculate that the selective inhibition of the SecYEG‐translocon by ES24 may cause the accumulation and aggregation of precursor forms of secreted proteins and membrane proteins that are known to induce IbpA/B upregulation (Kuczyńska‐Wiśnik *et al*., 2002). In short, our transcriptomics analysis suggests that reduced reactive intermediates of both NFT and ES24 non‐selectively interfere with a range of important cellular processes. However, in marked contrast to ES24, our data show that NFT does not affect SecYEG‐mediated protein translocation functions in *E. coli*. We, therefore, conclude that ES24 has specific structural features that may promote its binding to and inactivation of the SecYEG‐translocon, and indeed we find that minor structural changes hamper its effect on the SecYEG‐translocon.

How does ES24 affect the function of the bacterial and eukaryotic Sec‐translocon? Both share a three‐subunit core, with SecY and Sec61α forming the translocation channel in prokaryotes and eukaryotes, respectively (Rapoport *et al*., 2017). These subunits are comprised of 10 transmembrane helixes arranged in 2 halves and closed by the pore ring and plug domain when in the idle state (Van Den Berg *et al*., 2004). In the open state, SecY/Sec61α not only facilitates protein translocation across the membrane, the channel also opens up sideways at the so‐called lateral gate formed by TMD2 and 7 to allow the transfer of hydrophobic signal peptides/TMDs into the lipid bilayer (Van Den Berg *et al*., 2004). ES24 was recently shown to cause a Sec61α mediated leakage of Ca^2+^ from the ER with modeling studies suggesting that ES24 binds to the cytosolic end of the lateral gate, and thereby wedges it in an open state (Gamayun *et al*., 2019). Given the strong structural and functional homology between Sec61α and SecY (Denks *et al*., 2014), we consider it likely that ES24 targets SecY in a comparable manner. In support of this hypothesis, we show that NFT has no effect on ER Ca^2+^ homeostasis in mammalian cells consistent with the lack of effect of NFT on SecYEG‐mediated transport. Taken together, these data emphasize the importance of subtle structural features that define ES24 as a selective ligand for SecY/Sec61α. In view of the mandatory reduction of ES24 for it to be effective in live bacteria, it may well be that ES24 first undergoes reductive activation and subsequently one of the reduction products damages, among other targets, the SecYEG‐translocon. Since NfsA/B are located in the cytosol ES24 first has to cross the inner membrane before reductive activation, suggesting that ES24 interferes with SecYEG at the cytosolic side of the membrane. The small size of ES24 and its relative hydrophobicity may allow passive diffusion over the inner membrane.

Finally, ES24 showed potent anti‐bacterial activity against *E. coli* and other bacterial species including some belonging to the ESKAPE group of pathogens. The increased frequency of drug resistance among these pathogens necessitates the identification of novel antibiotics (Boucher *et al*., 2013). The repurposing of approved or investigational drugs for the treatment of bacterial infections has recently gained renewed interest (Miró‐canturri *et al*., 2019). Interestingly, ES1 was initially identified in a high‐throughput screen for small molecules that inhibit ERAD in eukaryotic cells (Fiebiger *et al*., 2004). Although its derivative ES24 retains the NFC‐domain that is typically avoided in most modern drug discovery programs as a known risk factor for cytotoxicity (Blass, 2015), NFT which contains an identical NFC‐domain, is widely used as an antibiotic to treat urinary tract infections (Panel and Chain, 2015). When delivered orally, most NFT is rapidly excreted into the urinary tract (Conklin, 1971; Wijma *et al*., 2018). As a result, the concentration of NFT in the urinary tract can exceed 200‐fold the plasma concentration, allowing the eradication of invading bacteria without damaging body tissue. Interestingly, ES24 showed MIC values against uropathogenic *E. coli* that are similar to NFT and was only mildly cytotoxic toward HEK293 cells in culture. This is in line with the low cytotoxicity of ES24 previously reported for HEK293 cells where it is effects appear to correlate with the sensitivity of different mammalian cell lines to perturbations of ER Ca^2+^ homeostasis (Gamayun *et al*., 2019). In contrast, ES1 was far more toxic for all mammalian cell lines tested (Gamayun *et al*., 2019).

Strikingly, when compared to NFT, ES24 shows enhanced anti‐bacterial activity toward various Gram‐positive bacteria, including the clinically relevant pathogens VRE and MRSA. These pathogens may also cause urinary tract infections, particularly in a hospital setting (Looney *et al*., 2017). Whether the increased sensitivity of Gram‐positive bacteria to ES24 is simply due to its increased accessibility to their Sec‐translocation machinery, or it is more effective for some other reason remains to be determined. Nevertheless, the broad and effective antibacterial activity of ES24 that we describe here, combined with its mild cytotoxicity in human cell lines warrant examination of the pharmacokinetics and therapeutic capacity of ES24 in animal models. In addition, ES24 may be used as a tool compound to manipulate SecYEG function in in vitro assays to study protein trafficking.

## 4 EXPERIMENTAL PROCEDURES

### 4.1 Strains, plasmids and media

The bacterial strains and plasmids that were used in this study are listed in Tables S1 and S2, respectively. The bacteria were grown in Luria Broth (LB) or in minimal M9 including 0.2% glucose and 0.2% casamino acids (Difco). For selective growth and transformations chloramphenicol (30 µg/ml), ampicillin (100 µg/ml), streptomycin (100 µg/ml), kanamycin (50 µg/ml), and tetracycline (12.5 µg/ml) were added to the medium, where appropriate. Clinical isolates were obtained from the Amsterdam University Medical Center, the Netherlands.

### 4.2 Materials, reagents, enzymes and sera

Growth in 96‐well plates was performed in µClear Chimney black clear‐bottom plates TC sterile from Greiner Bio‐One. Plates were sealed with non‐sterile clear multi‐well plate sealers from Greiner Bio‐One. Furagin was purchased from MedChemExpress. The compounds ES24‐D1 and ES24‐D2 were obtained from Mcule. All other reagents and chemicals were bought from Sigma–Aldrich. Stock solutions of all compounds were made by dissolving the compounds in DMSO. The compounds ES1 and ES24 were synthesized as previously described (McKibbin *et al*., 2012; Gamayun *et al*., 2019). Antisera against GFP, Hbp passenger domain and Hbp β‐domain were from our own collection. HRP‐conjugated affinity purified anti‐rabbit IgG from Rockland was used as the secondary antibody.

### 4.3 Susceptibility to antimicrobials

Growth experiments were performed in 96‐well plates. The relevant bacterial strains were first grown in LB to mid‐log phase in regular culture flasks at 37°C. The culture was then diluted to an optical density (OD) at 600 nm of 0.001 and 50 µl aliquots were transferred to a 96‐well plate that already contained 50 µl LB with a 2‐fold increasing concentration of compound including 200 µM as highest concentration (0.5% DMSO as final concentration). After sealing the plate, growth was continued at 37°C in the Synergy H1 plate reader (Biotek) with 3 mm continuous linear shaking. The OD_600_ was measured every 15 min for 18 hr. The MIC was determined after 18 hr of growth as minimum concentration where no growth of strains could be detected by eye.

### 4.4 Transcriptomics

*E. coli* MC4100 cells were grown to mid log phase in LB at 37°C in duplicate in regular culture flasks and diluted to an OD_600_ of 0.05 in 10 ml LB in a 50 ml round bottom tube. Subsequently, 0.5% DMSO, 12 µM ES24 or 12 µM NFT was added to the culture as final concentration followed by 20 min incubation with shaking at 37°C. The culture suspension was pelleted by centrifugation at 5,000× *g* for 5 min and used to extract total RNA using the Qiagen RNA purification kit according to the manufacturer's instructions. Transcriptomics and differential expression analysis were performed by Macrogen (Seoul, Korea).

### 4.5 Live cell calcium imaging

Ca^2+^ imaging experiments were carried out with the HEK‐D1ER cell line that expresses the FRET‐based Ca^2+^ Sensor D1ER (Gamayun *et al*., 2019). D1ER is a chameleon construct comprised of the fluorescent proteins CFP and citrine coupled by two calcium‐sensing proteins that undergo a conformational change upon binding of Ca^2+^. The FRET efficiency of the CFP‐citrine pair changes to maximum upon binding of Ca^2+^, while in a Ca^2+^ free environment FRET between CFP and citrine does not occur. D1ER was excited at 433 nm and the emitted fluorescence was then split at 469/23 nm and 536/27 nm to obtain the CFP and citrine components, respectively. CFP‐citrine image pairs containing 5–10 cells/frame were obtained every 10 s. D1ER ratios (DR) were calculated from CFP‐citrine image pairs as ratios of background‐subtracted citrine fluorescence at 536 nm to CFP fluorescence at 469 nm. D1ER ratios were subsequently normalized as (DR‐DR_min_)/(DR_max_‐DR_min_), where DR_max_ and DR_min_ represent the maximum and minimum of D1ER ratios measured in each cell.

HEK‐D1ER cells were maintained in culture under selection with G418 (0.5 mg/ml) in Minimal Essential Medium (MEM) supplemented with 10% (v/v) fetal bovine serum (Thermo Fisher Scientific) in a 5% CO_2_ humidified incubator at 37°C. Ca^2+^ imaging experiments were performed with cells that were plated on poly‐L‐lysine‐coated cover slips and cultured for 72 hr. In order to prevent the Ca^2+^ entry from the extracellular space, all Ca^2+^ imaging experiments were carried out in a Ca^2+^ free recording solution (140 mM NaCl, 4 mM KCl, 1 mM MgCl_2_, 0.5 mM EGTA, 10 mM Glucose, 10 mM HEPES, pH 7.4).

NFT, ES1, and ES24 were prepared freshly in DMSO just before the experiments and the compounds were further diluted to a 2× concentration in the Ca^2+^ free recording solution. Routinely, application of NFT and ES24 was achieved by adding 2× solutions to the recording chamber at a ratio of 1:1 to avoid problems arising from slow mixing. The maximal DMSO concentration in the recording chamber was 0.1% v/v.

Statistical significance of the Ca^2+^ imaging data was assessed with the two‐sample Kolmogorov‐Smirnov test. Statistical significance is given as n.s., nonsignificant; ***, *p* < .001.

### 4.6 Monitoring heat‐shock and cell envelope stress

*E. coli* TOP10F′ cells, harboring pUA66‐GroES‐mNG or pUA66‐RpoE‐mNG, were grown in M9 at 37°C in regular culture flasks to an OD_600_ of 0.5. The culture was then diluted to an OD_600_ of 0.1 and 50 µl aliquots were transferred to a 96‐well plate that already contained 50 µl M9 with compound or 0.5% DMSO per well as final concentration. Growth was continued at 37°C in the Synergy H1 plate reader with 3 mm continuous linear shaking. The OD_600_ and fluorescence (485/535 nm) was measured every 15 min for 3 hr.

### 4.7 Microscopy analysis

*E. coli* MC4100 cells, harboring pSE(p15a)‐NG‐WALP‐F, pSE(p15a)‐NG‐WALP‐F‐TolR, or pSE(p15a)‐NG, were grown in LB at 37°C in regular culture flasks to an OD_600_ of 0.5. The cultures were then diluted to an OD_600_ of 0.1 and 50 µl aliquots were transferred to a 96‐well plate that already contained 50 µl LB with 6 µM ES24, 6 µM NFT, or 0.5% DMSO per well as final concentration. The 96‐well plate was incubated with shaking for 1 hr at 37°C. Next, 80 µM IPTG was added as final concentration to induce protein expression from the plasmids followed by 1 hr incubation with shaking at 37°C. Subsequently, cells were pelleted by centrifugation at 5,000× *g* for 5 min and fixed with 4% of paraformaldehyde and 0.5% of glutaraldehyde in phosphate‐buffered saline (PBS) for 1 hr at 4°C, washed once with PBS and stored at 4°C in 100 µl PBS. For analysis, 2 µl of the bacterial suspension was immobilized on 1% Noble agar (w/v in PBS) pads and imaged with the Olympus IX83 using a 100×/N.A 1.35 oil objective and 500‐ms exposure time for the GFP channel. The software ImageJ and the ImageJ plug‐in ObjectJ, Coli‐counter and the CrossProfiles‐Macro1.0 were used to quantify membrane localization of the fluorescent reporter proteins as we described earlier (Peschke *et al*., 2019).

### 4.8 HEK293 cell toxicity

HEK293 cells were cultured in regular flasks at 37°C, 5% CO_2_ in DMEM medium supplemented with 10% FBS (Thermo Fisher Scientific) for multiple passages. Next, 50,000 cells were seeded in each well of a 96‐well plate (Corning, flat bottom, tissue culture treated) followed by overnight incubation at 37°C with 5% CO_2_. The next day fresh DMEM medium was added that already contained a twofold dilution range of compounds or DMSO as control (1% final concentration). Cells were incubated for an additional 24 hr before cell viability was measured using a resazurin assay (Borra *et al*., 2009).

### 4.9 Hbp secretion analysis

*E. coli* TOP10F′, *E. coli* AB1157, and *E. coli* NER502 ∆*nfsA/B* cells, harboring pEH3‐Hbp or pEH3‐GFP, were grown in LB to mid‐log phase in regular culture flasks at 37°C and diluted to an OD_600_ of 0.25 in 20 ml round bottom tubes already containing ES24, NFT, or DMSO as control. After 5 min of incubation with shaking at 37°C expression of Hbp and GFP from the pEH3 vector was induced with 80 µM IPTG as final concentration. After 10 and 30 min of incubation with shaking at 37°C 1 ml culture aliquots were withdrawn and cells were collected at 5,000× *g* for 10 min. The spent medium was precipitated with trichloroacetic acid (TCA). Cell and medium fractions were analyzed by SDS‐PAGE and Western blotting.

## CONFLICT OF INTERESTS

The authors declared no potential conflicts of interest with respect to the research, authorship, and/or publication of this article.

## AUTHOR CONTRIBUTION

M. Steenhuis, J. Luirink, S. High, H.G. Koch, and A. Cavalié designed the study.

M. Steenhuis, G.M. Koningstein, J. Oswald, S. O’Keefe, R.C. Whitehead, E. Swanton, and T. Pick acquired, analyzed, and interpreted the data.

M. Steenhuis and J. Luirink wrote the manuscript.

## Supporting information

Supplementary Material

## References

[mmi14589-bib-0001] Aletrari, M.O.O. , McKibbin, C. , Williams, H. , Pawar, V. , Pietroni, P. , Lord, J.M. , *et al*. (2011) Eeyarestatin 1 interferes with both retrograde and anterograde intracellular trafficking pathways. PLoS One, 6(7), e22713. 10.1371/journal.pone.0022713 21799938 PMC3143184

[mmi14589-bib-0002] Blass, B. (2015) Case studies in drug discovery. In Basic Principles of Drug Discovery and Development (1st edition). Elsevier Science Publishing Co Inc, 512–513.

[mmi14589-bib-0003] Borra, R.C. , Lotufo, M.A. , Gagioti, S.M. , de Barros, F.M. and Andrade, P.M. (2009) A simple method to measure cell viability in proliferation and cytotoxicity assays. Brazilian Oral Research, 23(3), 255–262. 10.1590/S1806-83242009000300006 19893959

[mmi14589-bib-0004] Boucher, H.W. , Talbot, G.H. , Benjamin, D.K. , Bradley, J. , Guidos, R.J. , Jones, R.N. , *et al*. (2013) 10 x ’20 progress‐development of new drugs active against gram‐negative Bacilli: an update from the infectious diseases society of America. Clinical Infectious Diseases, 56(12), 1685–1694. 10.1093/cid/cit152 23599308 PMC3707426

[mmi14589-bib-0005] Buchberger, A. , Bukau, B. and Sommer, T. (2010) Protein quality control in the cytosol and the endoplasmic reticulum: brothers in arms. Molecular Cell, 40(2), 238–252. 10.1016/j.molcel.2010.10.001 20965419

[mmi14589-bib-0006] Conklin, J. (1971) The pharmacokinetics of nitrofurantoin and its related bioavailability. Antimicrobial Agents and Chemotherapy, 25, 233–252. 10.1159/000401065 352255

[mmi14589-bib-0007] Cross, B.C.S. , McKibbin, C. , Callan, A.C. , Roboti, P. , Piacenti, M. , Rabu, C. , *et al*. (2009) Eeyarestatin I inhibits Sec61‐mediated protein translocation at the endoplasmic reticulum. Journal of Cell Science, 122(23), 4393–4400. 10.1242/jcs.054494 19903691 PMC2779136

[mmi14589-bib-0008] Denks, K. , Vogt, A. , Sachelaru, I. , Petriman, N.A. , Kudva, R. and Koch, H.G. (2014) The Sec translocon mediated protein transport in prokaryotes and eukaryotes. Molecular Membrane Biology, 31(2–3), 58–84. 10.3109/09687688.2014.907455 24762201

[mmi14589-bib-0009] Du Plessis, D.J.F. , Nouwen, N. and Driessen, A.J.M. (2011) The Sec translocase. Biochimica et Biophysica Acta ‐ Biomembranes, 1808(3), 851–865. 10.1016/j.bbamem.2010.08.016 20801097

[mmi14589-bib-0010] Fiebiger, E. , Hirsch, C. , Vyes, J. , Gordon, E. , Ploegh, H. and Tortorella, D. (2004) Dissection of the dislocation pathway for type i membrane proteins with a new small molecule inhibitor, eeyarestatin. Molecular Biology of the Cell, 15, 1635–1646. 10.1091/mbc.E03 14767067 PMC379262

[mmi14589-bib-0011] Gamayun, I. , Keefe, S.O. , Pick, T. , Klein, M. and Nguyen, D. (2019) Eeyarestatin compounds selectively enhance Sec61‐mediated Ca^2+^ leakage from the endoplasmic reticulum. Cell Chemical Biology, 26(4), 571–583. 10.1016/j.chembiol.2019.01.010. 30799222 PMC6483976

[mmi14589-bib-0012] Holt, A. and Killian, J.A. (2010) Orientation and dynamics of transmembrane peptides: the power of simple models. European Biophysics Journal, 39(4), 609–621. 10.1007/s00249-009-0567-1 20020122 PMC2841270

[mmi14589-bib-0013] Huttner, A. , Verhaegh, E.M. , Harbarth, S. , Muller, A.E. , Theuretzbacher, U. and Mouton, J.W. (2015) Nitrofurantoin revisited: a systematic review and meta‐analysis of controlled trials. Journal of Antimicrobial Chemotherapy, 70(9), 2456–2464. 10.1093/jac/dkv147 26066581

[mmi14589-bib-0014] Jong, W.S.P. , Saurí, A. and Luirink, J. (2010) Extracellular production of recombinant proteins using bacterial autotransporters. Current Opinion in Biotechnology, 21(5), 646–652. 10.1016/j.copbio.2010.07.009 20932460

[mmi14589-bib-0015] Krishnamoorthy, G. , Wolloscheck, D. , Weeks, J.W. , Croft, C. , Rybenkov, V.V. and Zgurskaya, H.I. (2016) Breaking the permeability barrier of *Escherichia coli* by controlled hyperporination of the outer membrane. Antimicrobial Agents and Chemotherapy, 60, 7372–7381. 10.1128/AAC.01882-16 27697764 PMC5119019

[mmi14589-bib-0016] Kuczyńska‐Wiśnik, D. , Kedzierska, S. , Matuszewska, E. , Lund, P. , Taylor, A. , Lipińska, B. , *et al*. (2002) The *Escherichia coli* small heat‐shock proteins IbpA and IbpB prevent the aggregation of endogenous proteins denatured *in vivo* during extreme heat shock. Microbiology, 148, 1757–1765. 10.1099/00221287-148-6-1757 12055295

[mmi14589-bib-0017] Lang, S. , Pfeffer, S. , Lee, P.H. , Cavalié, A. , Helms, V. , Förster, F. , *et al*. (2017) An update on Sec 61 channel functions, mechanisms, and related diseases. Frontiers in Physiology, 8, 1–22. 10.3389/fphys.2017.00887 29163222 PMC5672155

[mmi14589-bib-0018] Lemus, L. and Goder, V. (2014) Regulation of endoplasmic reticulum‐associated protein degradation (ERAD) by ubiquitin. Cells, 3(3), 824–847. 10.3390/cells3030824 25100021 PMC4197631

[mmi14589-bib-0019] Looney, A.T. , Redmond, E.J. , Davey, N.M. , Daly, P.J. , Troy, C. , Carey, B.F. , *et al*. (2017) Methicillin‐resistant *Staphylococcus aureus* as a uropathogen in an Irish setting. Medicine, 96(14), 1–5. 10.1097/MD.0000000000004635 PMC5411178 28383394

[mmi14589-bib-0020] McCalla, D.R. , Kaiser, C. and Green, M.H.L. (1978) Genetics of nitrofurazone resistance in *Escherichia coli* . Journal of Bacteriology, 133(1), 10–16. 10.1128/JB.133.1.10-16.1978 338576 PMC221970

[mmi14589-bib-0021] McKenna, M. , Simmonds, R. and High, S. (2016) Mechanistic insights into the inhibition of Sec61‐dependent co‐ and post‐ translational translocation by mycolactone. The Company of Biologists Ltd, 44, 4–8. 10.1242/jcs.179960 PMC4852723 26869228

[mmi14589-bib-0022] McKibbin, C. , Mares, A. , Piacenti, M. , Williams, H. , Roboti, P. , Puumalainen, M. , *et al*. (2012) Inhibition of protein translocation at the endoplasmic reticulum promotes activation of the unfolded protein response. The Biochemical Journal, 442(3), 639–648. 10.1042/BJ20111220 22145777 PMC3286858

[mmi14589-bib-0023] McOsker, C.C. , Fitzpatrick, P.M.P.M. , Mc Osker, C.C. and Fitzpatrick, P.M.P.M. (1994) Nitrofurantoin: mechanism of action and implications for resistance development in common uropathogens. Journal of Antimicrobial Chemotherapy, 33, 23–30. 10.1093/jac/33.suppl_A.23 7928834

[mmi14589-bib-0024] Miró‐canturri, A. , Ayerbe‐algaba, R. and Smani, Y. (2019) Drug repurposing for the treatment of bacterial and fungal infections. Frontiers in Immunology, 10, 1–12. 10.3389/fmicb.2019.00041 30745898 PMC6360151

[mmi14589-bib-0025] Niedzwiecki, D.J. , Mohammad, M.M. and Movileanu, L. (2012) Inspection of the engineered FhuA Δc/Δ4L protein nanopore by polymer exclusion. Biophysical Journal, 103(10), 2115–2124. 10.1016/j.bpj.2012.10.008 23200045 PMC3512039

[mmi14589-bib-0026] Nikaido, H. (2003) Molecular basis of bacterial outer membrane permeability revisited. Microbiology and Molecular Biology Reviews, 67(4), 593–656. 10.1128/MMBR.67.4.593 14665678 PMC309051

[mmi14589-bib-0027] Palmer, A.E. , Jin, C. , Reed, J.C. and Tsien, R.Y. (2004) Bcl‐2‐mediated alterations in endoplasmic reticulum Ca2+ analyzed with an improved genetically encoded fluorescent sensor. PNAS, 101(50), 17404–17409. 10.1073/pnas.0408030101 15585581 PMC535104

[mmi14589-bib-0028] Panel, E. and Chain, F. (2015) Scientific opinion on nitrofurans and their metabolites in food. EFSA Journal, 13(6), 1–217. 10.2903/j.efsa.2015.4140

[mmi14589-bib-0029] Peschke, M. , Le Goff, M. , Koningstein, G.M. , Vischer, N.O. , Abdel‐rehim, A. , High, S. , *et al*. (2019) Distinct requirements for tail‐anchored membrane protein biogenesis in *Escherichia coli* . MBio, 10(5), 1–18. 10.1128/mBio.01580-19 PMC6794478 31615956

[mmi14589-bib-0030] Rapoport, T.A. , Li, L. and Park, E. (2017) Structural and mechanistic insights into protein translocation. Annual Review of Cell and Developmental Biology, 33(1), 369–390. 10.1146/annurev-cellbio-100616-060439 28564553

[mmi14589-bib-0031] Ruggiano, A. , Foresti, O. and Carvalho, P. (2014) ER‐associated degradation: protein quality control and beyond. Journal of Cell Biology, 204(6), 869–879. 10.1083/jcb.201312042 PMC3998802 24637321

[mmi14589-bib-0032] Sandegren, L. , Lindqvist, A. , Kahlmeter, G. and Andersson, D.I. (2008) Nitrofurantoin resistance mechanism and fitness cost in *Escherichia coli* . Journal of Antimicrobial Chemotherapy, 62(3), 495–503. 10.1093/jac/dkn222 18544599

[mmi14589-bib-0033] Sannino, S. and Brodsky, J.L. (2017) Targeting protein quality control pathways in breast cancer. BMC Biology, 15(109), 1–20. 10.1186/s12915-017-0449-4 29145850 PMC5689203

[mmi14589-bib-0034] Sauri, A. , Soprova, Z. , Wickström, D. , de Gier, J.W. , Van der Schors, R.C. , Smit, A.B. , *et al*. (2009) The Bam (Omp85) complex is involved in secretion of the autotransporter haemoglobin protease. Microbiology, 155(Pt 12), 3982–3991. 10.1099/mic.0.034991-0 19815580

[mmi14589-bib-0035] Sijbrandi, R. , Urbanus, M.L. , Ten Hagen‐Jongman, C.M. , Bernstein, H.D. , Oudega, B. , Otto, B.R. , *et al*. (2003) Signal recognition particle (SRP)‐mediated targeting and Sec‐dependent translocation of an extracellular *Escherichia coli* protein. Journal of Biological Chemistry, 278(7), 4654–4659. 10.1074/jbc.M211630200 12466262

[mmi14589-bib-0036] Steenhuis, M. , Abdallah, A.M. , De Munnik, S.M. , Kuhne, S. , Westerhausen, S. , Wagner, S. , *et al*. (2019) Inhibition of autotransporter biogenesis by small molecules. Molecular Microbiology, 112(1), 1–18. 10.1111/mmi.14255 30983025 PMC6850105

[mmi14589-bib-0037] Tu, Y. and McCalla, D.R. (1975) Effect of activated nitrofurans on DNA. BBA Section Nucleic Acids and Protein Synthesis, 402(2), 142–149. 10.1016/0005-2787(75)90032-5 1100114

[mmi14589-bib-0038] Valle, A. , Borgne, S.L. and Bolívar, J. (2012) Study of the role played by NfsA, NfsB nitroreductase and NemA flavin reductase from *Escherichia coli* . Applied Microbiology and Biotechnology, 94, 163–171. 10.1007/s00253-011-3787-0 22173483

[mmi14589-bib-0039] Van Den Berg, B. , Clemons, W.M. , Collinson, I. , Modis, Y. , Hartmann, E. , Harrison, S.C. , *et al*. (2004) X‐ray structure of a protein‐conducting channel. Nature, 427(6969), 36–44. 10.1038/nature02218 14661030

[mmi14589-bib-0040] van Ulsen, P. , Rahman, S.U. , Jong, W.S.P. , Daleke‐Schermerhorn, M.H. and Luirink, J. (2013) Type V secretion: from biogenesis to biotechnology. Biochimica et Biophysica Acta, 1843, 1592–1611. 10.1016/j.bbamcr.2013.11.006 24269841

[mmi14589-bib-0041] Wang, Q. , Li, A. and Ye, Y. (2008) Inhibition of p97‐dependent protein degradation by Eeyarestatin I. Journal of Biological Chemistry, 283(12), 7445–7454. 10.1074/jbc.M708347200 PMC2276333 18199748

[mmi14589-bib-0042] Wang, Q. , Shinkre, B.A. , Lee, J.G. , Weniger, M.A. , Liu, Y. , Chen, W. , *et al*. (2010) The ERAD inhibitor eeyarestatin I is a bifunctional compound with a membrane‐binding domain and a p97/VCP inhibitory group. PLoS One, 5(11), e15479. 10.1371/journal.pone.0015479 21124757 PMC2993181

[mmi14589-bib-0043] Whiteway, J. , Koziarz, P. , Veall, J. , Sandhu, N. , Kumar, P. , Hoecher, B. , *et al*. (1998) Oxygen‐insensitive nitroreductases: analysis of the roles of nfsA and nfsB in development of resistance to 5‐nitrofuran derivatives in *Escherichia coli* . Journal of Bacteriology, 180(21), 5529–5539. 10.1128/JB.180.21.5529-5539.1998 9791100 PMC107609

[mmi14589-bib-0044] Wijma, R.A. , Huttner, A. , Koch, B.C.P. , Mouton, J.W. and Muller, A.E. (2018) Review of the pharmacokinetic properties of nitrofurantoin and nitroxoline. Journal of Antimicrobial Chemotherapy, 73(11), 2916–2926. 10.1093/jac/dky255 30184207

