# Supplementary material for: Eeyarestatin 24 impairs SecYEG‐dependent protein trafficking and inhibits growth of clinically relevant pathogens
Source: Mol Microbiol. 2020 Sep 3;115(1):28–40. doi: 10.1111/mmi.14589 (PMC8273874; doi:10.1111/mmi.14589)
Supplement: Supplementary file 1 — Supplementary Material [file MMI-115-28-s001.pdf]

Supporting information

Eeyarestatin 24 Impairs SecYEG-dependent Protein Trafficking and Inhibits Growth of Clinically Relevant Pathogens

Maurice Steenhuis[1], Gregory M. Koningstein[1], Julia Oswald[2], Tillman Pick[3], Sarah O'Keefe[4], Hans-Georg Koch[2], Adolfo Cavalié[3], Roger C. Whitehead[5], Eileithyia Swanton[4], Stephen High[4] and Joen Luirink[1,*]

[1]Department of Molecular Microbiology, Amsterdam Institute of Molecular and Life Sciences (AIMMS), Vrije Universiteit, Amsterdam, the Netherlands

[2]Institute of Biochemistry and Molecular Biology, Faculty of Medicine, University of Freiburg, Freiburg, Germany

[3]Experimental and Clinical Pharmacology and Toxicology, Saarland University, 66421 Homburg, Germany

[4]School of Biological Sciences, Faculty of Biology, Medicine and Health, University of Manchester, Manchester, M13 9PT, United Kingdom

[5]School of Chemistry, Faculty of Science and Engineering, University of Manchester, Manchester, M13 9PL, United Kingdom

*Corresponding author
Joen Luirink, Molecular Microbiology, Vrije Universiteit, De Boelelaan 1085, 1081 HV Amsterdam, the Netherlands. Email: s.luirink@vu.nl. Tel: +31 (0)20 598 7175

Keywords
Escherichia coli, nitrofurantoin, Sec61, eeyarestatin 1

Contents

Figure S1. Differential effect of ES24, ES1 and NFT on various strains. 3

Figure S2. ES24 impairs membrane insertion of NG-WALP-F-TolR, but not of NG-WALP-F. 4

Figure S3. NG expression is not affected by ES24 nor NFT. 5

Figure S4. Differential effect of ES24 and NFT on heat-shock stress induction. 6

Figure S5. Differential effect of ES24-derivatives, NFT and Furagin on growth of *E. coli.* 7

Figure S6. Furagin, ES24–D1 and ES24–D2 do not interfere with membrane localization of NG-WALP-F-TolR and NG-WALP-F. 8

Figure S7. Differential effect of ES24 and NFT on various strains. 9

Figure S8. Differential effect of ES1, ES24 and NFT on growth of *B. subtilis.* 10

Table S1. List of bacterial strains used in this study. 11

Table S2. List of plasmids used in this study. 11

Table S3. Up-regulated and down-regulated genes (≥3 fold) in *E. coli* upon incubation with ES24 or NFT compared to DMSO-treated cells. 12

References 17

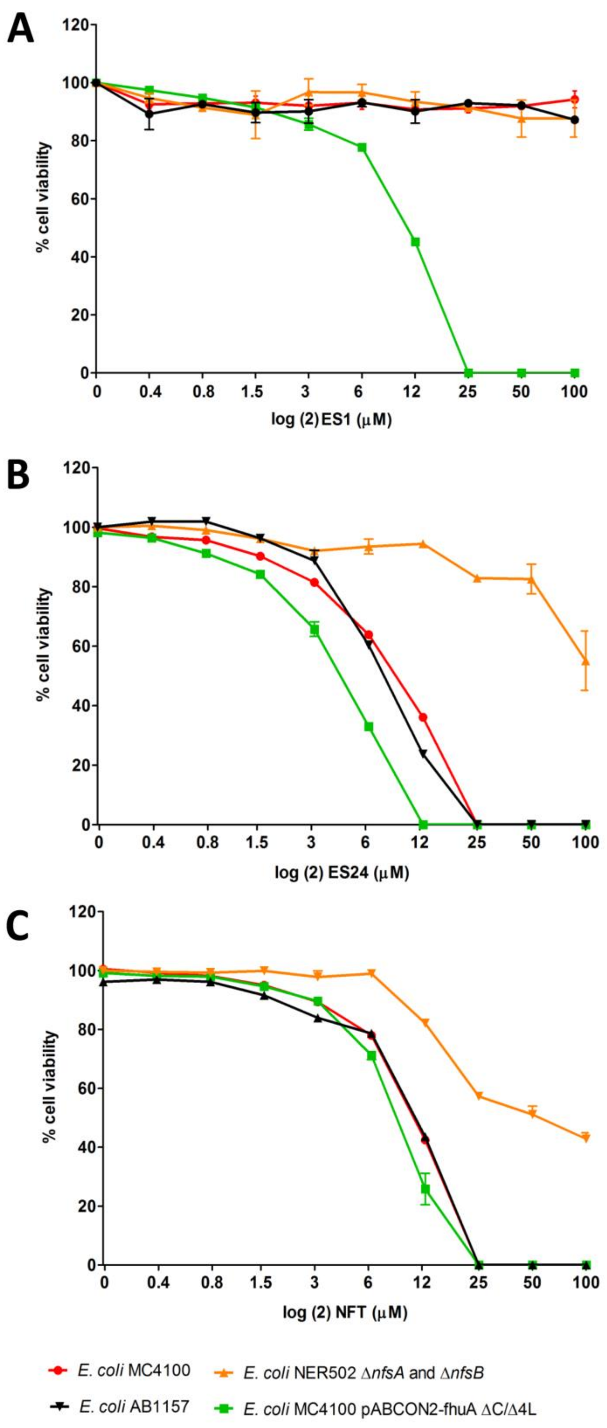

Figure S1. Effects of ES24, ES1 and NFT on growth of mutant strains.
The indicated bacterial strains were grown in LB in a 96-well plate and incubated with a two-fold increasing concentration of **(A)** ES1, **(B)** ES24 or **(C)** NFT. Growth was continued for 18 hours and plotted as percentage cell viability compared to untreated cells (set to 100%). Error bars indicate the standard deviation of triplicate samples.

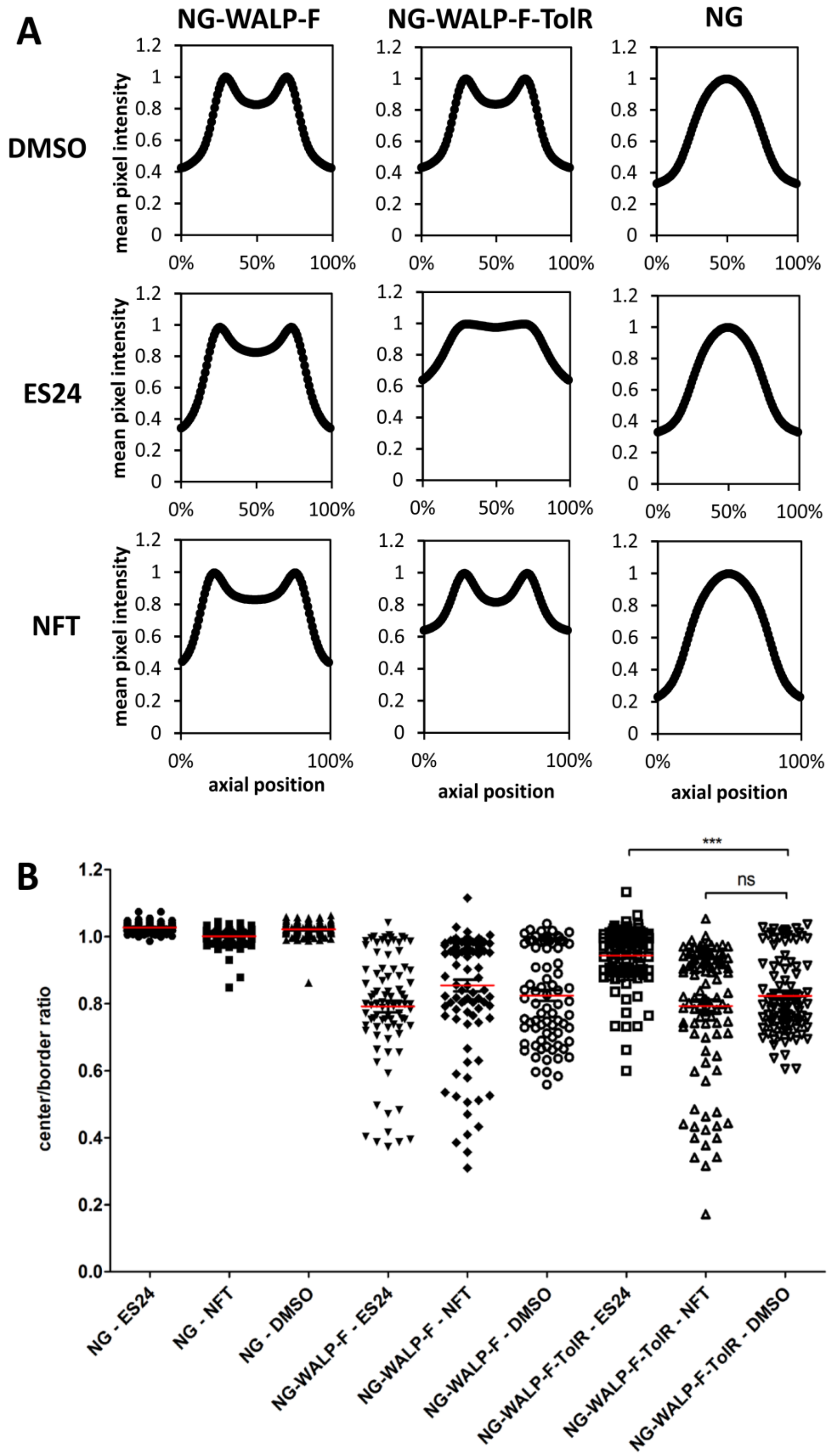

Figure S2. ES24 impairs membrane insertion of NG-WALP-F-TolR, but not of NG-WALP-F.
(A) Individual cells were selected and manually confirmed after the spatial distribution of fluorescence perpendicular to the cell axis was measured, based on the images in Fig. 2B. The plotted distribution along the cell axis (axial position) per condition is an average of 100 individual cells. Data are representative of two independent experiments. **(B)** Center/border ratios of the fluorescence signal were calculated from cross profiles (see Fig. 2 and S2A). The red line represents the median. Multiple comparisons among groups were carried out by One-Way ANOVA and Tukey's post-test (Prism software). ns: non-significant; *** $p < 0.005$.

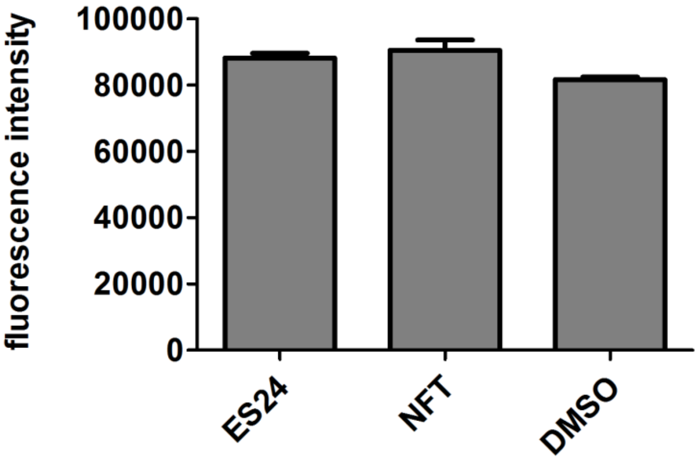

Figure S3. NG expression is not affected by ES24 nor by NFT.
The fluorescent intensity of *E. coli* MC4100 cells expressing NG from pSE(p15a) was measured for each condition indicated in Fig. 2, with the synergy H1 plate reader at the same time point when cells were withdrawn for fluorescence microscopy analysis. Error bars represent the standard deviation of triplicate samples**.**

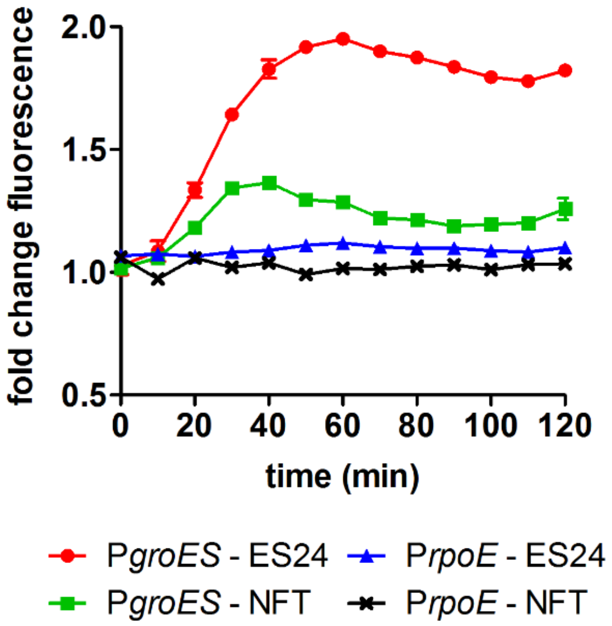

Figure S4. Effect of ES24 and NFT on heat-shock stress induction.
E. coli TOP10F’ cells were grown in M9 in a 96-well plate and incubated with 12 µM ES24, 12 µM NFT or DMSO as control. Intracellular heat-shock stress and the σ^E cell envelope stress response was monitored in time using the pUA66-GroES-mNG and pUA66-RpoE-mNG reporter constructs, respectively. The fold change fluorescence was compared to DMSO-treated cells (set to 1). Error bars represent the standard deviation of triplicate samples.

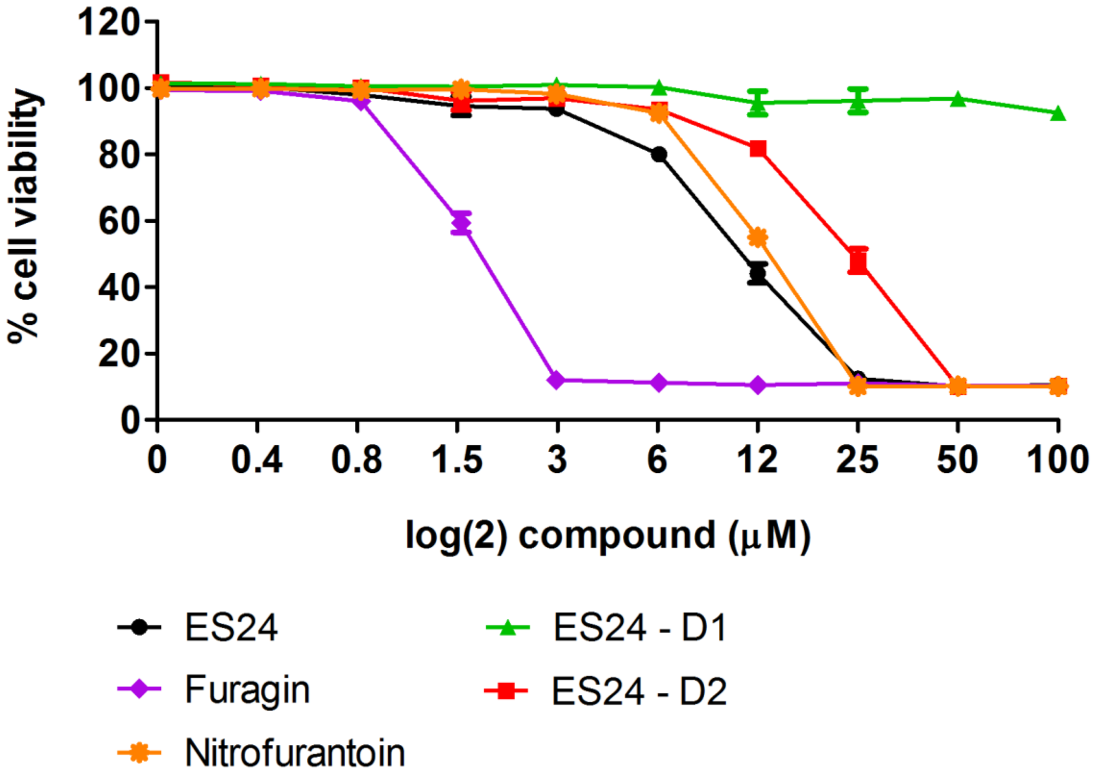

Figure S5. Effect of ES24-derivatives, NFT and Furagin on growth of *E. coli.*

E. coli TOP10F' cells were grown in LB in a 96-well plate and incubated with a two-fold increasing concentration of the indicated compounds. Growth was continued for 18 h and plotted as percentage cell viability compared to untreated cells (set to 100%). Error bars indicate the standard deviation of triplicate samples.

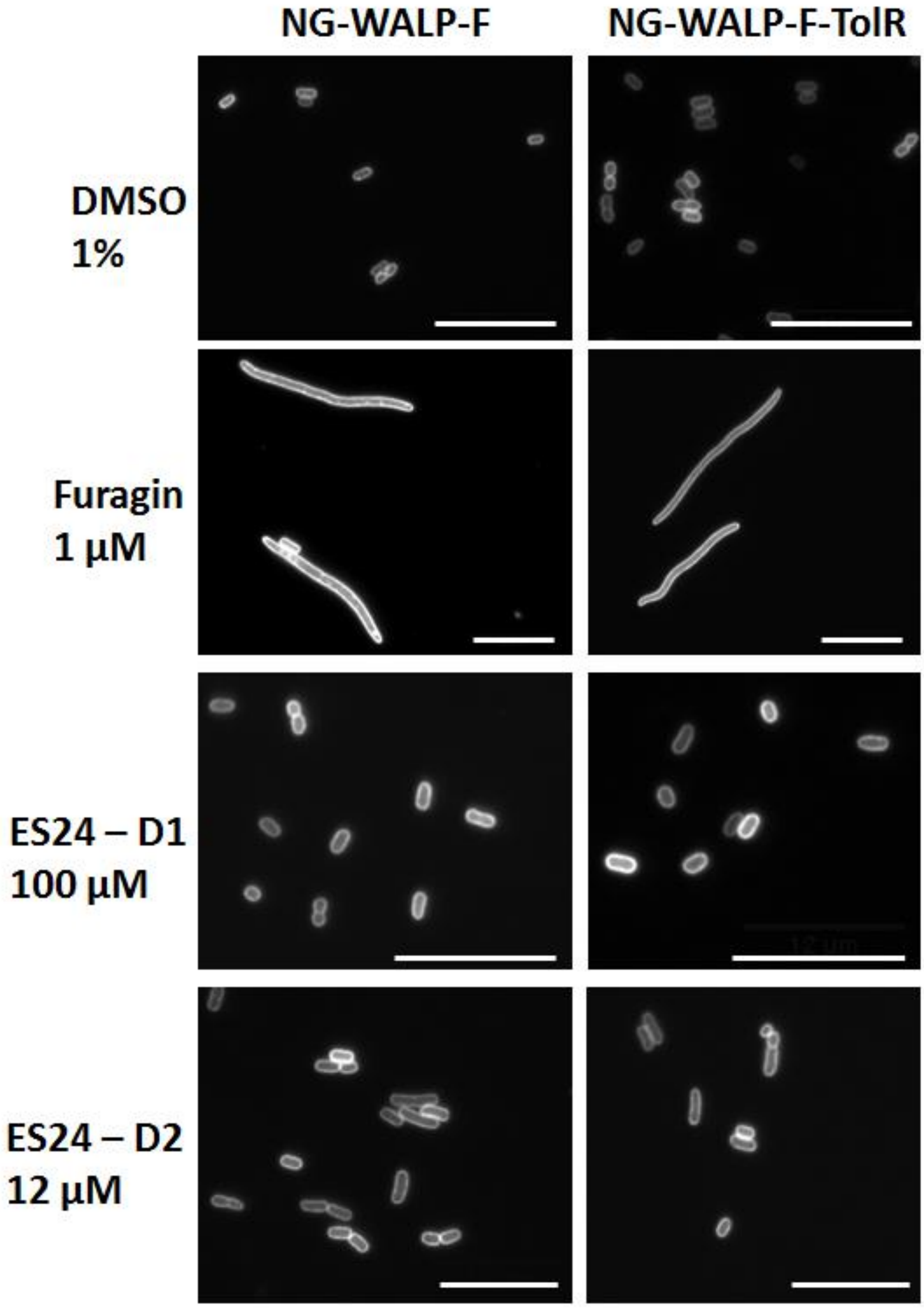

Figure S6. Furagin, ES24–D1 and ES24–D2 do not interfere with membrane localization of NG-WALP-F-TolR and NG-WALP-F.

E. coli MC4100 cells, harboring pSE(p15a)-NG-WALP-F or pSE(p15a)-NG-WALP-F-TolR, were grown in a 96-well plate and incubated for 1 h with the indicated compounds. Next, IPTG was added to induce protein expression from the plasmids followed by 1 h incubation before cells were fixed with formaldehyde and analyzed by fluorescence microscopy. Scale bars are 12 µm. Data are representative of two independent experiments.

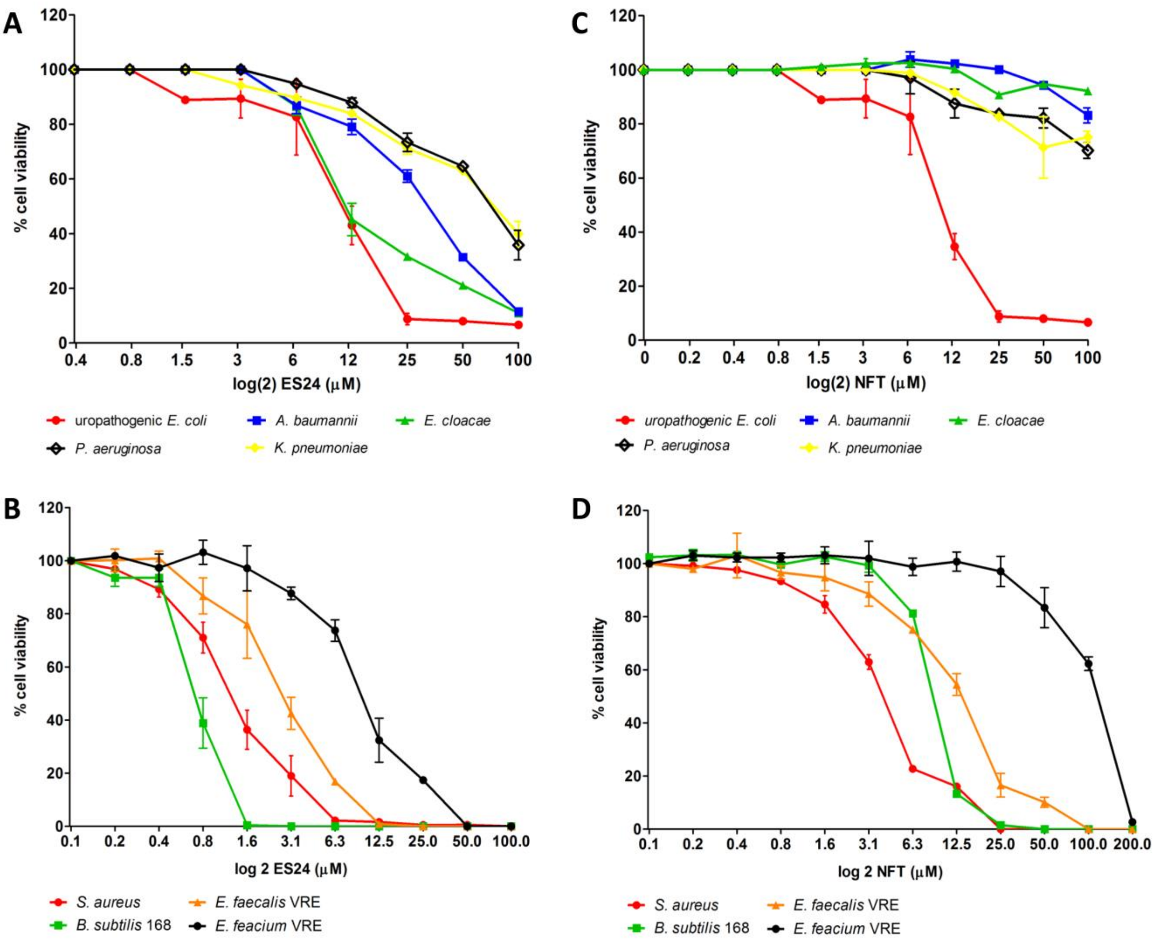

Figure S7. Effect of ES24 and NFT on growth of various Gram-negative and Gram-positive species. The Gram-negative **(A, C)** and Gram-positive **(B, D)** species were grown in LB in a 96-well plate and incubated with a two-fold increasing concentration of ES24 or NFT. Growth was continued for 18 hours and plotted as percentage cell viability compared to untreated cells (set to 100%). Error bars indicate the standard deviation of triplicate samples.

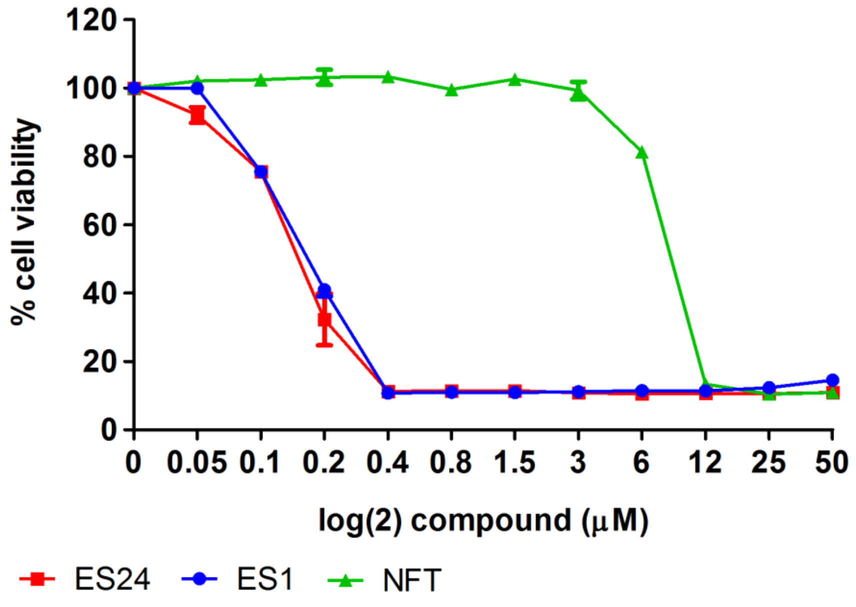

Figure S8. Effect of ES1, ES24 and NFT on growth of *B. subtilis.*
B. subtilis 168 cells were grown in LB in a 96-well plate and incubated with a two-fold increasing concentration of the indicated compounds. Growth was determined by measuring the OD_{600} after 18 h of growth and plotted as % cell viability with the DMSO control set to 100%. Error bars represent the standard deviation of triplicate samples.

Table S1. List of bacterial strains used in this study.

Bacterial strain	Description	Reference
E. coli MC4100	Cloning and expression strain	(Taschner, Huls, Pas, & Woldringh, 1988)
E. coli TOP10F'	Cloning and expression strain	Thermo Fisher Scientific
E. coli AB1157	Parental strain NER502	(González-Pérez, Van Dillewijn, Wittich, & Ramos, 2007)
E. coli NER502 Δ*nfsA/B*	Deletion of the nitroreductases *nfsA* and *nfsB*	(González-Pérez *et al*., 2007)
Enterococcus faecium (VRE)	Vancomycin-resistant clinical isolate	This study
Enterococcus faecalis (VRE)	Vancomycin-resistant clinical isolate	This study
Staphlycoccus aureus (MRSA)	Methicillin-resistant clinical isolate	This study
Klebsiella pneumoniae	Clinical isolate	This study
Acinetobacter baumannii	Clinical isolate	This study
Pseudomonas aeruginosa	Clinical isolate	This study
Enterobacter cloacae	Clinical isolate	This study
Escherichia coli	Uropathogenic clinical isolate	This study

Table S2. List of plasmids used in this study.

Plasmid name	Description	Reference
pSE(p15a)-His-NG-WALP-F	P_{lac}-*neongreen-WALP*-His_6 (F)	(Peschke *et al*., 2019)
pSE(p15a)-NG-WALP-F-TolR	P_{lac}-*neongreen-WALP-tolR*-His_6 (F)	(Peschke *et al*., 2019)
pSE(p15a)-NG	P_{lac}-*neongreen*	(Peschke *et al*., 2019)
pABCON2-fhuA ΔC/Δ4L	P_{OXB11}-*fhuA ΔC/Δ4L*	(Jonkers *et al*., 2020)
pUA66-GroES-mNG	P_{groES}-*neongreen*	(Steenhuis *et al*., 2019)
PUA66-RpoE-mNG	P_{rpoE}-*neongreen*	(Steenhuis *et al*., 2019)
pEH3	Expression vector; *lac*UV5 promoter	(Hashemzadeh-Bonehi *et al*., 1998)
pEH3-Hbp	P_{lac}-*hbp*	(Jong *et al*., 2007)
pEH3-GFP	P_{lac}-*gfp*	(Yu *et al*., 2011)

Table S3. Up-regulated and down-regulated genes (≥3 fold) in *E. coli* upon incubation with ES24 or NFT compared to DMSO-treated cells.

Gene ID[a]	Gene	Description/function[a]	Log_2 fold change NFT/DMSO[b]	Log_2 fold change ES24/DMSO[b]
	genes under control of σ^{32} factor (heat-shock response)			
BWG_2352	*clpB*	protein disaggregation chaperone	11	22
BWG_0320	*cplX*	ATP-dependent protease ATP-binding subunit	-	3
BWG_0014	*dnaJ*	chaperone protein DnaJ	23	36
BWG_0013	*dnaK*	molecular chaperone DnaK	34	58
BWG_3853	*fxsA*	FxsA, unknown function	13	33
BWG_3540	*glnA*	glutamine synthetase	-	-3
BWG_3856	*groEL*	chaperonin GroEL	21	34
BWG_3855	*groES*	co-chaperonin GroES	17	31
BWG_2372	*grpE*	heat shock protein GrpE	5	9
BWG_3092	*hslO*	Hsp33-like chaperonin	5	10
BWG_3091	*hslR*	ribosome-associated heat shock protein Hsp	7	12
BWG_3600	*hslU*	ATP-dependent protease ATP-binding subunit	6	12
BWG_3601	*hslV*	ATP-dependent protease peptidase subunit	5	12
BWG_0354	*htpQ*	heat shock protein 90	13	25
BWG_3377	*ibpA*	heat shock protein IbpA	21	81
BWG_3376	*ibpB*	heat shock chaperone IbpB	27	410
BWG_0321	*lon*	DNA-binding ATP-dependent protease	5	8
BWG_0223	*phoE*	outer membrane phosphoporin protein E	-5	-4
BWG_0373	*ybbN*	putative thioredoxin domain-containing protein	3	5
BWG_0531	*ybeZ*	hypothetical protein	3	4
BWG_1504	*ydiM*	putative transporter	-4	-
	genes under control of LexA and RecA (SOS-response)			
BWG_1554	*cho*	nucleotide excision repair endonuclease	6	5
BWG_0215	*dinB*	DNA polymerase IV	5	6
BWG_3336	*dinD*	DNA-damage-inducible protein D	3	4
BWG_3757	*dinF*	DNA-damage-inducible SOS response protein	3	4
BWG_0652	*dinG*	ATP-dependent DNA helicase DinG	3	3
BWG_4040	*dinL*	endoribonuclease SymE	7	9
BWG_3756	*lexA*	LexA repressor	3	-
BWG_2419	*nrdF*	ribonucleotide-diphosphate reductase subunit	23	10
BWG_2416	*nrdH*	glutaredoxin-like protein	24	12
BWG_0056	*polB*	DNA polymerase II	4	4
BWG_2435	*recA*	recombinase A	6	21
BWG_2374	*recN*	recombination and repair protein	15	8
BWG_2434	*recX*	recombination regulator RecX	5	7
BWG_0810	*sulA*	SOS cell division inhibitor	11	13
BWG_1009	*umuC*	DNA polymerase V subunit UmuC	9	10
BWG_1008	*umuD*	DNA polymerase V subunit UmuD	7	8
BWG_3772	*uvrA*	excinuclease ABC subunit A	5	6
BWG_0632	*uvrB*	excinuclease ABC subunit B	3	4
BWG_0654	*ybiC*	hypothetical protein	14	9
BWG_0655	*ybiJ*	hypothetical protein	14	43
BWG_0657	*ybiX*	putative hydroxylase	5	-
BWG_1319	*ydeN*	hypothetical protein	-	-3
BWG_1541	*ydjM*	hypothetical protein	3	4
BWG_1642	*yebQ*	putative transporter	5	7

BWG_2671	*ruvX*	holliday junction resolvase-like protein	4	3
	genes under control of OxyR (oxidative stress response)			
BWG_3901	*bsmA*	putative biofilm stress and motility	-3	-4
BWG_0702	*grxA*	glutaredoxin 1	4	8
BWG_3611	*katG*	catalase/hydroperoxidase HPI(I)	3	5
BWG_3507	*metE*	5-methyltetrahydropteroyltriglutamate-homocysteine	-3	-3
BWG_1498	*sufA*	iron-sulfur cluster assembly scaffold protein	7	3
BWG_1497	*sufB*	cysteine desulfurase activator complex subunit	7	3
BWG_1496	*sufC*	cysteine desulfurase ATPase component	6	3
BWG_1495	*sufD*	cysteine desulfurase activator complex subunit	7	3
BWG_1493	*sufE*	cysteine desufuration protein SufE	6	3
BWG_1494	*sufS*	bifunctional cysteine desulfurase/selenocy	7	3
	genes under control of SoxS, SoxR, Rob and MarA regulon (oxidative stress response)			
BWG_1107	*acnA*	aconitate hydratase	4	3
BWG_2912	*arcB*	aerobic respiration control sensor protein	3	-
BWG_3593	*fpr*	ferredoxin-NADP reductase	4	3
BWG_1426	*fumC*	fumarate hydratase	8	6
BWG_1882	*gatY*	tagatose-bisphosphate aldolase	-3	-3
BWG_2010	*inaA*	hypothetical protein	13	6
BWG_0902	*lpxL*	lipid A biosynthesis lauroyl acyltransferase	5	4
BWG_1350	*marA*	DNA-binding transcriptional activator MarA	11	9
BWG_1351	*marB*	hypothetical protein	10	9
BWG_1349	*marR*	DNA-binding transcriptional repressor MarR	5	4
BWG_2741	*mdaB*	NADPH quinone reductase	6	4
BWG_0330	*mdlA*	putative multidrug transporter membraneATP	10	3
BWG_0331	*mdlB*	putative multidrug transporter membraneATP	10	3
BWG_0724	*poxB*	pyruvate dehydrogenase	9	4
BWG_0705	*rimK*	ribosomal protein S6 modification protein	12	7
BWG_3578	*sodA*	superoxide dismutase	5	4
BWG_3399	*tnaA*	tryptophanase	5	13
BWG_2748	*tolC*	outer membrane channel protein	3	3
BWG_1206	*ydbK*	fused putative pyruvate-flavodoxin oxidore	5	4
	genes involved in iron uptake, Fe-S systems and thiol-redox			
BWG_1937	*cirA*	colicin I receptor	3	-
BWG_2726	*dkgA*	2,5-diketo-D-gluconate reductase A	3	3
BWG_0469	*entA*	2,3-dihydroxybenzoate-2,3-dehydrogenase	5	-
BWG_0468	*entB*	isochorismatase	4	-
BWG_0466	*entC*	isochorismate synthase 1	3	-
BWG_0455	*entD*	phosphopantetheinyltransferase	3	-
BWG_0467	*entE*	enterobactin synthase subunit E	4	-
BWG_0459	*entF*	enterobactin synthase subunit F	5	-
BWG_0470	*entH*	hypothetical protein	5	-
BWG_3099	*feoA*	ferrous iron transport protein A	-3	-4
BWG_0456	*fepA*	outer membrane receptor FepA	4	-
BWG_0457	*fes*	enterobactin/ferric enterobactin esterase	4	-
BWG_0950	*fhuE*	ferric-rhodotorulic acid outer membrane receptor	3	-
BWG_1717	*ftnA*	ferritin	-3	-
BWG_1714	*ftnB*	putative ferritin-like protein	-6	-7
BWG_2429	*gshA*	glutamate-cysteine ligase	3	3
BWG_2669	*gshB*	glutathione synthetase	5	4

BWG_1981	*napF*	ferredoxin-type protein	-7	-4
BWG_3586	*sbp*	sulfate transporter subunit	-	-3
BWG_0458	*ybdZ*	hypothetical protein	5	-
BWG_1277	*yncD*	putative iron outer membrane transporter	8	7
BWG_2780	*yqjH*	putative siderophore interacting protein	6	3
	genes involved in cysteine synthesis and transport proteins			
BWG_3192	*arsB*	arsenite/antimonite transporter	3	-
BWG_1782	*cbL*	transcriptional regulator Cbl	-3	-3
BWG_2184	*cysA*	sulfate/thiosulfate transporter subunit	-12	-4
BWG_2486	*cysC*	adenylylsulfate kinase	-9	-6
BWG_2488	*cysD*	sulfate adenylyltransferase subunit 2	-13	-11
BWG_2498	*cysH*	phosphoadenosine phosphosulfate reductase	-6	-
BWG_2500	*cysJ*	sulfite reductase subunit alpha	-17	-14
BWG_2487	*cysN*	sulfate adenylyltransferase subunit 1	-6	-3
BWG_2187	*cysP*	thiosulfate transporter subunit	-17	-26
BWG_2186	*cysU*	sulfate/thiosulfate transporter subunit	-14	-18
BWG_2185	*cysW*	sulfate/thiosulfate transporter permease	-14	-17
BWG_3161	*tusA*	sulfur transfer protein SirA	-5	-
BWG_3034	*tusB*	sulfur transfer complex subunit TusB	3	6
BWG_2818	*yhaM*	hypothetical protein	14	-
BWG_2819	*yhaO*	putative transporter	8	-
	genes related to reductases			
BWG_1239	*azoR*	azoreductase	3	25
BWG_1465	*nemA*	N-ethylmaleimide reductase	6	10
BWG_0704	*nemR*	nitroreductase A	6	3
BWG_0449	*nfsA*	dihydropteridine reductase	15	9
BWG_1239	*nfsB*	azoreductase	6	4
	genes involved in arginine metabolism			
BWG_2553	*argA*	N-acetylglutamate synthase	-4	-5
BWG_3627	*argB*	acetylglutamate kinase	-3	-6
BWG_3626	*argC*	N-acetyl-gamma-glutamyl-phosphate reductase	-6	-16
BWG_4166	*argQ*	Arg tRNA	-4	-7
BWG_0713	*artJ*	arginine transporter subunit	-3	-6
	genes related to the outer membrane proteins and LPS			
BWG_1989	*ompC*	outer membrane porin protein C	-5	-5
BWG_0781	*ompF*	outer membrane protein F	-11	-10
BWG_1085	*ompW*	outer membrane protein W	-6	-4
	genes related to other stress responses			
BWG_0154	*degP*	serine endoprotease	-3	-3
BWG_1136	*pspA*	phage shock protein PspA	-3	-9
BWG_1137	*pspB*	phage shock protein B	-4	-14
BWG_1138	*pspC*	DNA-binding transcriptional activator PspC	-3	-13
BWG_1139	*pspD*	peripheral inner membrane phage-shock protein	-4	-20
BWG_1140	*pspE*	thiosulfate	-3	-7
BWG_3763	*pspG*	phage shock protein G	-3	-12
BWG_0960	*bhsA*	hypothetical protein	37	53
	genes involved in efflux pumps biosynthesis			
BWG_0199	*gloB*	hydroxyacylglutathione hydrolase	3	-
BWG_1847	*wcaC*	putative glycosyl transferase	3	3
BWG_0148	*yadQ*	chloride channel protein	6	4
BWG_0121	*yadH*	putative transporter subunit	6	4

BWG_2171	*yfeH*	putative inner membrane protein	3	3
	genes related to fimbria and flagella			
BWG_0920	*flgA*	flagellar basal body P-ring biosynthesis protein	3	3
BWG_1743	*fliF*	flagellar MS-ring protein	3	3
BWG_1745	*fliH*	flagellar assembly protein H	4	-
BWG_1748	*fliK*	flagellar hook-length control protein	4	5
BWG_1749	*fliL*	flagellar basal body-associated protein FliL	3	4
BWG_1751	*fliN*	flagellar motor switch protein FliN	4	6
BWG_1753	*fliP*	flagellar biosynthesis protein FliP	3	3
BWG_4018	*fimH*	minor component of type 1 fimbriae	-3	-3
	genes expressing the cold-shock proteins			
BWG_1376	*cspB*	Qin prophage; cold shock protein	-	-3
BWG_1636	*cspC*	cold shock-like protein CspC	-	-3
BWG_0843	*cspG*	cold shock protein CspG	-	-3
BWG_0842	*cspH*	stress protein, member of the CspA-family	-4	-4
BWG_3244	*cspA*	major cold shock protein	-3	-9
	genes related to protein synthesis			
BWG_3186	*rsmJ*	putative methyltransferase	4	6
BWG_2668	*rsmE*	16S ribosomal RNA methyltransferase RsmE	4	3
	others			
BWG_2843	*agaC*	PTS system N-acetylgalactosamine-specific	-	-3
BWG_0856	*agP*	glucose-1-phosphatase/inositol phosphatase	-	-3
BWG_2147	*alaC*	aminotransferase	3	-
BWG_3852	*aspA*	aspartate ammonia-lyase	-5	-5
BWG_0689	*bssR*	biofilm formation regulatory protein BssR	-5	-4
BWG_1925	*cdd*	cytidine deaminase	-3	-3
BWG_0959	*comR*	putative DNA-binding transcriptional regulator	3	4
BWG_0365	*copA*	copper exporting ATPase	-	11
BWG_0491	*citC*	citrate lyase synthetase	3	-
BWG_0443	*cusC*	copper/silver efflux system outer membrane	4	-
BWG_0471	*cstA*	carbon starvation protein	3	-
BWG_3217	*dctA*	C4-dicarboxylate transporter DctA	-3	-3
BWG_1728	*dcyD*	D-cysteine desulfhydrase	3	-
BWG_1355	*dgcZ*	hypothetical protein	-	-3
BWG_0658	*fiu*	catecholate siderophore receptor Fiu	4	-
BWG_2539	*fucP*	L-fucose transporter	-10	-7
BWG_2833	*garP*	putative (D)-galactarate transporter	-5	-5
BWG_0117	*gcd*	glucose dehydrogenase	4	3
BWG_2013	*glpA*	n-glycerol-3-phosphate dehydrogenase	-9	-11
BWG_2014	*glpB*	anaerobic glycerol-3-phosphate dehydrogenase	-3	-3
BWG_2012	*glpT*	n-glycerol-3-phosphate transporter	-14	-10
BWG_3128	*gntK*	gluconate kinase 1	-6	-4
BWG_3127	*gntU*	low affinity gluconate transporter	-3	-3
BWG_0256	*hemB*	delta-aminolevulinic acid dehydratase	7	5
BWG_2461	*hycA*	formate hydrogenlyase regulatory protein	-4	-5
BWG_2247	*hyfC*	hydrogenase 4, membrane subunit	3	-
BWG_2614	*idi*	isopentenyl-diphosphate delta-isomerase	13	10
BWG_3974	*idnK*	D-gluconate kinase	-	-3
BWG_3579	*kdgT*	2-keto-3-deoxygluconate permease	3	3
BWG_3112	*malT*	transcriptional regulator MalT	-3	-3

BWG_1630	*manX*	fused mannose-specific PTS enzymes	-7	-5
BWG_3831	*melR*	DNA-binding transcriptional regulator MelR	-7	-7
BWG_2322	*mltF*	putative transglycosylase	3	3
BWG_0900	*msyB*	hypothetical protein	3	4
BWG_0602	*nadA*	quinolinate synthetase	-	-3
BWG_2338	*nadB*	L-aspartate oxidase	-	-9
BWG_2231	*narQ*	nitrate/nitrite sensor protein NarQ	-10	-9
BWG_1051	*narK*	nitrate/nitrite transporter	-15	-12
BWG_3167	*nikA*	nickel transporter subunit	-4	-3
BWG_3057	*nirB*	nitrite reductase, large subunit, NAD(P)H-	-4	-4
BWG_1710	*otsB*	trehalose-6-phosphate phosphatase	3	-
BWG_0447	*pheP*	phenylalanine transporter	-4	-3
BWG_2691	*pppA*	bifunctional prepilin leader peptidase	-3	-4
BWG_3187	*prlC*	oligopeptidase A	4	6
BWG_3440	*rbsA*	D-ribose transporter ATP binding protein	-8	-6
BWG_3439	*rbsD*	D-ribose pyranase	-5	-5
BWG_3576	*rhaR*	transcriptional activator RhaR	-3	-3
BWG_1944	*rihB*	ribonucleoside hydrolase 2	7	6
BWG_2438	*srlA*	glucitol/sorbitol-specific enzyme IIC	-	-4
BWG_2826	*tdcA*	DNA-binding transcriptional activator TdcA	-16	-11
BWG_1416	*tqsA*	putative transport protein	-	-3
BWG_0850	TorC	trimethylamine N-oxide (TMAO) reductase	121	98
BWG_0851	TorA	trimethylamine N-oxide (TMAO) reductase	35	28
BWG_0852	TorD	chaperone protein TorD	17	13
BWG_3948	*treB*	PTS system trehalose(maltose)-specific transporter	-10	-9
BWG_3509	*udP*	uridine phosphorylase	-	-3
BWG_3144	*ugpB*	glycerol-3-phosphate transporter periplasm	-	-3
BWG_1204	*uspF*	stress-induced protein, ATP-binding protein	-4	-3
BWG_0477	*ybdO*	putative DNA-binding transcriptional regulator	9	-
BWG_1030	*ychH*	hypothetical protein	-5	-5
BWG_1087	*yciF*	hypothetical protein	-	-3
BWG_1141	*ycjM*	putative glucosyltransferase	-3	-3
BWG_1143	*ycjO*	putative sugar transporter subunit	-	-3
BWG_1361	*ydfZ*	hypothetical protein	-3	-3
BWG_1909	*yehU*	putative sensory kinase in two-component	-3	-3
BWG_2623	*ygfZ*	putative global regulator	4	3
BWG_2701	*yghQ*	putative inner membrane protein	-6	-3
BWG_2939	*yhcN*	hypothetical protein	6	-
BWG_3071	*yhfW*	putative mutase	4	-
BWG_3378	*yidQ*	hypothetical protein	-3	-3
BWG_3542	*yihL*	putative DNA-binding transcriptional regulator	-3	-3
BWG_1105	*ymiA*	hypothetical protein	-4	-3
BWG_1410	*ynfM*	putative transporter	3	-4
BWG_1637	*yobF*	hypothetical protein	-	-3
BWG_3160	*zntA*	zinc/cadmium/mercury/lead-transporting	-4	-4
BWG_3201	*gadE*	DNA-binding transcriptional activator	-	-3
BWG_0706	*otsB*	putative oxidoreductase	3	-
BWG_2816	*ybjN*	putative pirin-related protein	5	4
BWG_1803	*YhaK*	putative inner membrane protein	6	30
BWG_2725	*yeeE*	alcohol dehydrogenase, NAD(P)-dependent	-10	-5
BWG_3160	*yqhD*	zinc/cadmium/mercury/lead-transporting	4	4

[a]Gene ID and gene description are based on PromBase (http://nucleix.mbu.iisc.ernet.in-/prombase/index.htm)

[b]The log (base 2) changes indicate the ratios of gene signal intensities of the *E. coli* strain TOP10F' treated with 12 µM ES24 or 12 µM nitrofurantoin (NFT) for 15 min compared to DMSO-treated cells (1% as final concentration).

References

González-Pérez, M. M., Van Dillewijn, P., Wittich, R. M., & Ramos, J. L. (2007). *Escherichia coli* has multiple enzymes that attack TNT and release nitrogen for growth. *Environmental Microbiology*, *9*(6), 1535–1540. https://doi.org/10.1111/j.1462-2920.2007.01272.x

Hashemzadeh-Bonehi, L., Mehraein-Ghomi, F., Mitsopoulos, C., Jacob, J. P. J., Hennessey, E. S., Broome-Smith, J. K., … Broome-Smith, J. (1998). Importance of using lac rather than ara promoter vectors for modulating the levels of toxic gene products in *Escherichia coli*. *Molecular Microbiology*, *30*(3), 676–678.

Jong, W. S. P. P., Ten Hagen-Jongman, C. M., den Blaauwen, T., Slotboom, D. J., Tame, J. R. H. H., Wickström, D., De Gier, J.W., Otto, B.R and Luirink, J. (2007). Limited tolerance towards folded elements during secretion of the autotransporter Hbp. *Molecular Microbiology*, *63*(5), 1524–1536. https://doi.org/10.1111/j.1365-2958.2007.05605.x

Jonkers, T. J. H., Steenhuis, M., Schalkwijk, L., Luirink, J., Bald, D., Houtman, C. J., Kool, J., Lamoree, M.H and Hamers, T. (2020). Development of a high-throughput bioassay for screening of antibiotics in aquatic environmental samples. *Science of the Total Environment*, *729*, 139028. https://doi.org/10.1016/j.scitotenv.2020.139028

Peschke, M., Le Goff, M., Koningstein, G. M., Vischer, N. O., Abdel-rehim, A., High, S., Van Ulsen, P and Luirink, J. (2019). Distinct requirements for tail-anchored membrane protein biogenesis in *Escherichia coli*. *MBio*, *10*(5), 1–18. https://doi.org/10.1128/mBio.01580-19

Steenhuis, M., Abdallah, A. M., Munnik, S. M. De, Kuhne, S., Westerhausen, S., Wagner, S., Wagner, S., Van der Wel, N.N., Wijtmans, M., Van Ulsen, P., Jong, W.S.P and Luirink, J. (2019). Inhibition of autotransporter biogenesis by small molecules. *Molecular Microbiology*, *112*(1), 1–18. https://doi.org/10.1111/mmi.14255

Taschner, P. E., Huls, P. G., Pas, E., & Woldringh, C. L. (1988). Division behavior and shape changes in isogenic ftsZ, ftsQ, ftsA, pbpB, and ftsE cell division mutants of *Escherichia coli* during temperature shift experiments. *Journal of Bacteriology*, *170*(4), 1533–1540. https://doi.org/10.1128/jb.170.4.1533-1540.1988

Yu, Z., Lavèn, M., Klepsch, M., de Gier, J. W., Bitter, W., van Ulsen, P., & Luirink, J. (2011). Role for *Escherichia coli* YidD in membrane protein insertion. *Journal of Bacteriology*, *193*(19), 5242–5251. https://doi.org/10.1128/JB.05429-11
